# Proteasome inhibition by bortezomib parallels a reduction in head and neck cancer cells growth, and an increase in tumor-infiltrating immune cells

**DOI:** 10.1038/s41598-021-98450-6

**Published:** 2021-09-24

**Authors:** Monica Benvenuto, Sara Ciuffa, Chiara Focaccetti, Diego Sbardella, Sara Fazi, Manuel Scimeca, Grazia Raffaella Tundo, Giovanni Barillari, Maria Segni, Elena Bonanno, Vittorio Manzari, Andrea Modesti, Laura Masuelli, Massimo Coletta, Roberto Bei

**Affiliations:** 1 Saint Camillus International, University of Health and Medical Sciences, Via di Sant’Alessandro 8, 00131 Rome, Italy; 2 Department of Clinical Sciences and Translational Medicine, University of Rome “Tor Vergata”, Via Montpellier 1, 00133 Rome, Italy; 3 Department of Human Science and Promotion of the Quality of Life, San Raffaele University Rome, Via di Val Cannuta 247, 00166 Rome, Italy; 4 IRCCS-Fondazione Bietti, Rome, Italy; 5 Department of Experimental Medicine, University of Rome “Sapienza”, Viale Regina Elena 324, 00161 Rome, Italy; 6 Department of Experimental Medicine, University of Rome “Tor Vergata”, Via Montpellier 1, 00133 Rome, Italy; 7 Department of Maternal Infantile and Urological Sciences, University of Rome “Sapienza”, Viale Regina Elena 324, 00161 Rome, Italy; 8 Pediatric Endocrinology Unit, Policlinico Umberto I, Viale Regina Elena 364, 00161 Rome, Italy; 9 “Diagnostica Medica” & “Villa Dei Platani”, Neuromed Group, 83100 Avellino, Italy

**Keywords:** Cancer therapy, Targeted therapies

## Abstract

Head and neck cancer (HNC) has frequently an aggressive course for the development of resistance to standard chemotherapy. Thus, the use of innovative therapeutic drugs is being assessed. Bortezomib is a proteasome inhibitor with anticancer effects. In vitro antitumoral activity of Bortezomib was investigated employing human tongue (SCC-15, CAL-27), pharynx (FaDu), salivary gland (A-253) cancer cell lines and a murine cell line (SALTO-5) originated from a salivary gland adenocarcinoma arising in BALB-*neu*T male mice transgenic for the oncogene neu. Bortezomib inhibited cell proliferation, triggered apoptosis, modulated the expression and activation of pro-survival signaling transduction pathways proteins activated by ErbB receptors and inhibited proteasome activity in vitro. Intraperitoneal administration of Bortezomib delayed tumor growth of SALTO-5 cells transplanted in BALB-*neu*T mice, protracted mice survival and adjusted tumor microenvironment by increasing tumor-infiltrating immune cells (CD4^+^ and CD8^+^ T cells, B lymphocytes, macrophages, and Natural Killer cells) and by decreasing vessels density. In addition, Bortezomib modified the expression of proteasome structural subunits in transplanted SALTO-5 cells. Our findings further support the use of Bortezomib for the treatment of HNC and reveal its ineffectiveness in counteracting the activation of deregulated specific signaling pathways in HNC cell lines when resistance to proteasome inhibition is developed.

## Introduction

Head and neck cancer (HNC) is the seventh leading cause of cancer-related morbidity and mortality, with 890,000 new cases diagnosed every year^1,2^. HNC involves several anatomical sites including pharynx, larynx, oral cavity, tongue and salivary glands^2^ and often has an aggressive course with emerging resistance to conventional chemotherapy. Salivary gland carcinomas represent 6–8% of HNC, with heterogeneous morphologies and clinical outcomes^3^. The development of new targeted therapies has opened new scenarios for the treatment of solid malignancies^4,5^. Therefore, the use of new therapeutic agents is being evaluated in HNC as well^6–9^. Current targeted regimen for HNC patients employs epidermal growth factor receptor (EGFR) inhibitor(s) and the monoclonal antibody cetuximab^1,10^. However, despite promising outcomes in preclinical studies, resistance to anti-EGFR targeted therapies in clinical settings represents a limitation for prolonged use of these drugs^7,11,12^.

Among different targeted therapies, inhibitors of the Ubiquitin Proteasome System (UPS), and in particular of the proteasome, are assuming particular importance^13^. They have become a new relevant class of drugs for treatment of tumors, regulation of the immune response and as anti-inflammatory agents^14,15^.

The UPS is a major intracellular proteolytic pathway^16^. The substrate, usually short-lived proteins, is conjugated to a chain of at least four ubiquitin (Ub) moieties by E1-E2-E3 enzymes and then delivered to the proteasome, a multi-catalytic assembly, for its enzymatic processing^17^. In the canonical structural configuration, the proteasome is made up by the 19S RP (i.e., Regulatory Particle) which carries out the recognition of poly-ubiquitinated substrates (poly-Ub) and couples their ATP-dependent unfolding with the translocation into the catalytic chamber of the 20S CP (i.e., Core Particle)^15,17^. Thereafter, the substrate is digested through three enzymatic activities (i.e., chymotrypsin-like, trypsin-like and caspase-like, housed by the β5, β2 and β1 subunits, respectively)^18^. Mature 20S and 19S particles can assemble into different structural configurations, which display an emerging metabolic relevance since they are thought to deal with clearance of different subsets of natural substrates; in particular, poly-ubiquitinated proteins are fed to capped assemblies, whereas oxidized, misfolded or natively unfolded proteins are cleared by the 20S CP^17,19^. Nonetheless, the 20S/capped assemblies ratio has been uncovered to be finely tuned depending on the metabolic need of the cell and every living cell displays proteasome assemblies in the most suited configuration to cope with the maintenance of the proteostasis network (PN). PN defines the equilibrium between protein synthesis and degradation which must be kept unaltered over the cell life cycle to guarantee cell viability and proliferation. Given the central relevance of the UPS in regulating this key network, several pharmacological strategies have been undertaken over the last decades to design drugs which specifically target proteasome proteolytic activity. The purpose of proteasome inhibition is to block the degradation of altered proteins performed by this complex, stimulating the formation of a toxic environment which ultimately triggers the apoptotic processes. As a matter of fact, the use of proteasome inhibitors (PI) in oncology is constantly gaining more interest, as emphasized by the development of next generation proteasome inhibitors. Some of them has already entered clinical trials also in solid malignancies providing outstanding outcomes in terms of overall survival (OS)^15^. In this framework, the clinical success of Bortezomib in treatment of haematological disorders, in particular multiple myeloma, has pioneristically validated the relevance of this therapeutic strategy. Bortezomib ([(1*R*)-3-methyl-1-({(2*S*)-3-phenyl-2-[(pyrazin-2-ylcarbonyl)amino]propanoyl}amino)butyl]boronic acid) is a dipeptidyl boronic acid reversible inhibitor of the chymotrypsin-like activity of the 20S proteasome^14,20,21^. Bortezomib was first synthesized in 1995 and subsequently approved in 2003 as a preferential treatment for multiple myeloma^22^. Bortezomib has been reported to show a potent anticancer activity, both in vitro and in vivo, also in other cancer cell lines, like prostate cancer, pancreatic cancer, renal cell carcinoma and squamous cell carcinoma^23–26^. In addition, several clinical trials evaluated the effect of Bortezomib in combination with chemotherapy or other targeted therapies in HNC^27–34^.

The aim of our research was to evaluate the in vitro and in vivo antitumoral activity of Bortezomib in HNC. We analyzed the in vitro effects of Bortezomib on cell proliferation, cell death, cell cycle regulation, apoptosis, autophagy, expression and activation of several proteins involved in pro-survival signaling pathways in human and mouse HNC cell lines, including salivary gland cancer cell lines. We also evaluated the effects of Bortezomib in inhibiting the proteasome activity in individual HNC cell lines and correlated for the first time this effect with the modulation of different signal transduction pathways involved in head and neck neoplastic transformation. In addition, we explored for the first time the in vivo effects of Bortezomib in mice transplanted with murine adenocarcinoma salivary gland SALTO-5 cell line and analyzed tumor immune infiltrate (CD4^+^ and CD8^+^ T cells, B lymphocytes, macrophages, and Natural Killer (NK) cells), tumor vessels density and proteasome structural subunits (PSMA4, PSMD4, PSME1) and ubiquitin expression. Our study therefore provides additional information on the antitumoral effect of Bortezomib and on the development of resistance mechanisms associated with the activation of deregulated specific signaling pathways in HNC cell lines.

## Results

### Inhibition of tongue (SCC-15, CAL-27), pharynx (FaDu), and salivary gland (A-253, SALTO-5) cancer cells survival and death by Bortezomib

The survival of tongue (SCC-15, CAL-27), pharynx (FaDu), and salivary gland (A-253) cancer cells was evaluated by the Sulforhodamine B (SRB) assay after exposure to increasing doses of Bortezomib (6.25–12.5–25–50–100 nM) or vehicle control (DMSO) for 24, 48 and 72 h. The effect of Bortezomib was also analyzed in SALTO-5 cells to establish whether this mouse cell line was susceptible to the in vitro anti-tumoral activity of Bortezomib and thus it could be used as transplantable tumor cell line in BALB-*neu*T mice to determine the in vivo anti-tumoral effect of the drug.

**Figure 1:**
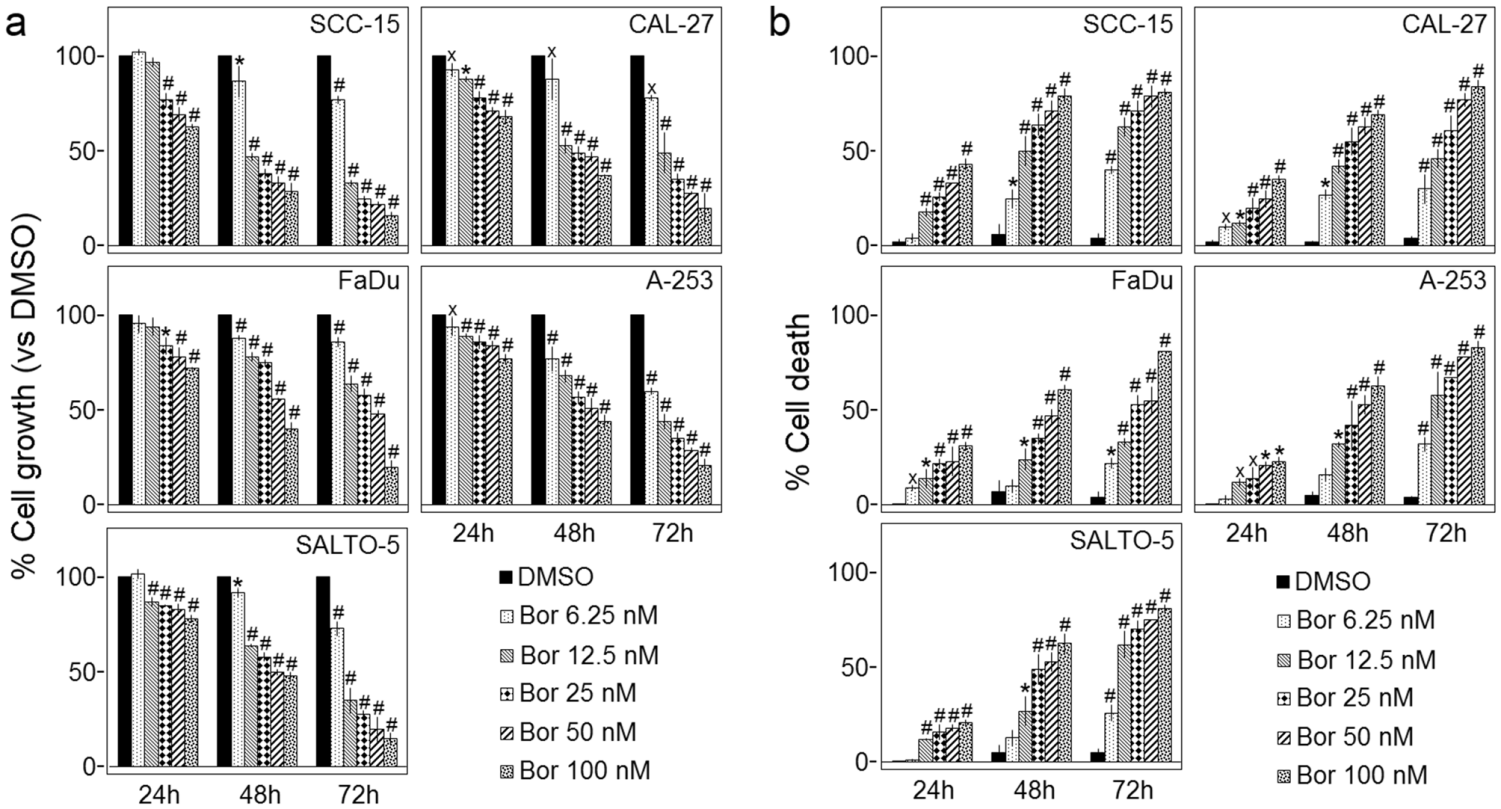
Effect of Bortezomib on survival of carcinoma cell lines of the tongue (SCC-15, CAL-27), pharynx (FaDu), and salivary gland (A-253, SALTO-5). (**a**) The growth rate of human (SCC-15, CAL-27, FaDu, A-253) and murine (SALTO-5) HNC cell lines was assessed by the SRB assay after 24, 48 and 72 h of treatment with DMSO or Bortezomib (Bor). The growth rate of cells treated with Bortezomib was calculated in comparison with that of control cells treated with DMSO. The results are expressed as the mean ± standard deviation (SD) of three independent experiments performed in triplicate. (˟*p* ≤ 0.05, *p ≤ 0.01, #*p* ≤ 0.001 *vs* DMSO). (**b**) The Trypan Blue assay was performed to determine the death rate of HNC cells treated with Bortezomib (Bor) or DMSO after 24, 48 and 72 h of treatment. Results represent the mean ± SD of three independent experiments performed in triplicate (˟*p* ≤ 0.05, *p ≤ 0.01, #*p* ≤ 0.001 *vs* DMSO).

The effect of Bortezomib on cell proliferation was dose- and time-dependent as compared to control cells. The greatest effect was obtained at the concentrations of 25, 50 and 100 nM and after 48 and 72 h of treatment (Fig. 1a). SCC-15 were the most sensitive cells to the drug's effect.

The death of human (SCC-15, CAL-27, FaDu, A-253) and mouse (SALTO-5) HNC cells was evaluated by the Trypan blue exclusion assay after exposure to increasing doses of Bortezomib (6.25–100 nM) or vehicle control (DMSO) for 24, 48 and 72 h. The dye exclusion assay was used to determine the number of viable HNC cells upon Bortezomib exposure. Bortezomib significantly increased the percentage of cell death in a dose- and time-dependent manner in all cell lines as compared to vehicle control after 24, 48 and 72 h (Fig. 1b).

In addition, the concentration of Bortezomib that inhibited 50% of cell growth (IC_50_) after 48 and 72 h was also determined. FaDu cells were the most resistant to Bortezomib activity, while SCC-15 was the most sensitive cell line (Table 1).

**Table 1:** Half maximal inhibitory concentration (IC_50_) of Bortezomib in inhibiting cell growth of SCC-15, CAL-27, FaDu, A-253 and SALTO-5 cell lines after 48 and 72 h of treatment.

Cell line	Treatment (hours)	IC_50_ ± SD (nM)
SCC-15	48	19.89 ± 3.46
	72	11.04 ± 0.26
CAL-27	48	33.73 ± 1.91
	72	15.97 ± 3.61
FaDu	48	67.83 ± 5.45
	72	32.92 ± 2.26
A-253	48	55.90 ± 19.01
	72	10.25 ± 1.50
SALTO-5	48	60.65 ± 7.83
	72	10.79 ± 1.84

### Effects of Bortezomib on apoptosis and cell cycle distribution of HNC cell lines

**Figure 2:**
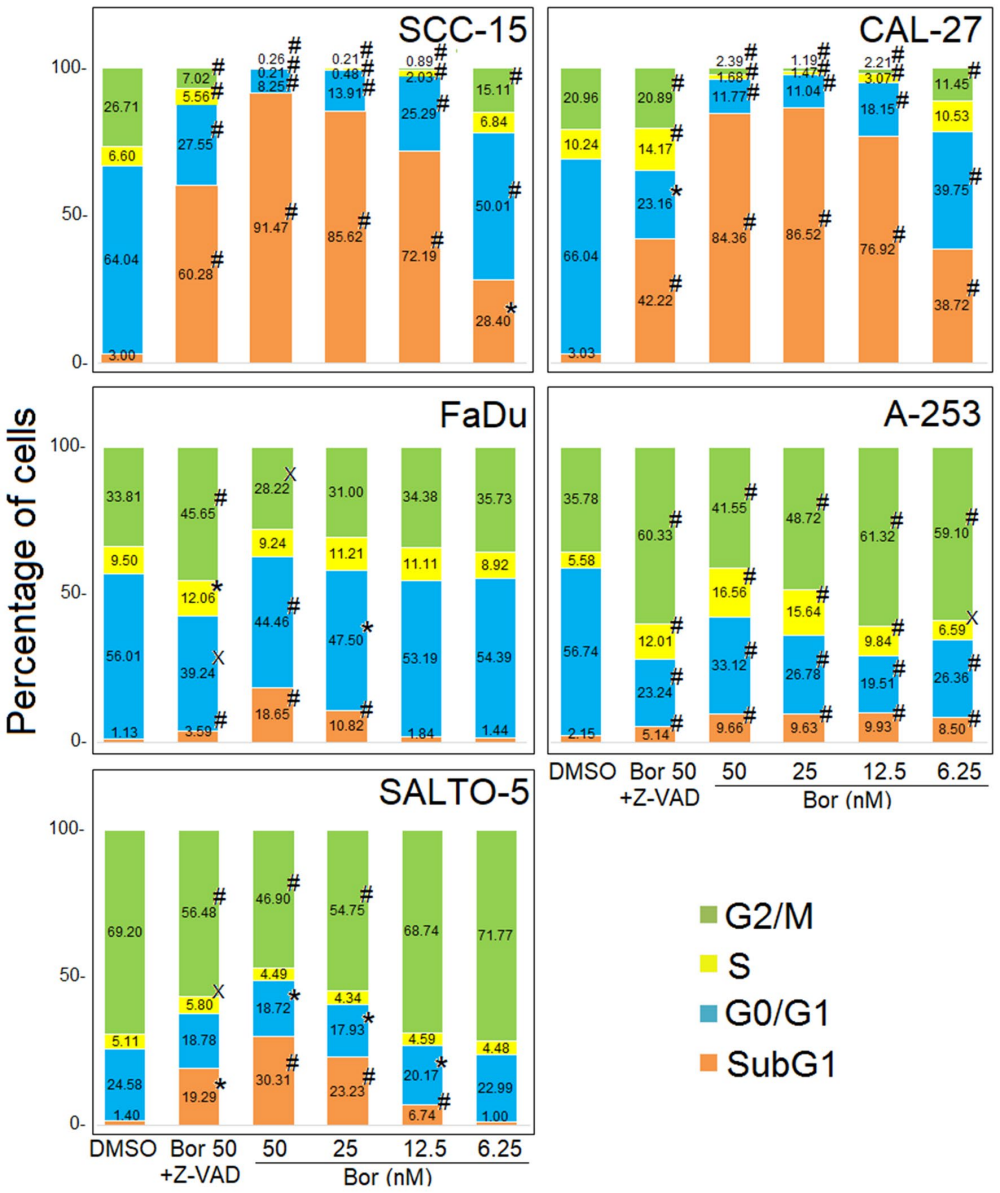
Stacked bar graphs showing the percentage of cells in different phases of the cell cycle. Percentage of cells in subG1, G0/G1, S, and G2/M phases was calculated with CellQuest Pro 5.2 software. Results represent the mean value of two experiments. The statistical significance of the effect obtained with the treatment with Bortezomib (Bor) was calculated with respect to that obtained with the vehicle alone (DMSO). The statistical significance of the effect obtained with Bortezomib at a dose of 50 nM together with the Z-VAD-FMK inhibitor (Bor50 + Z-VAD) was calculated with respect to that obtained in cells treated with Bortezomib at a dose of 50 nM (˟*p* ≤ 0.05, *p ≤ 0.01, #*p* ≤ 0.001).

To evaluate the effects of Bortezomib on apoptosis and cell cycle distribution, FACS analysis of DNA content was performed on HNC cells treated with increasing doses of Bortezomib (6.25–50 nM) for 48 h; DMSO was used as control vehicle. Our results showed that treatment with Bortezomib significantly increased the percentage of cells in the subG1 phase at all doses in SCC-15, CAL-27 and A-253 cells, at 25–50 nM in the FaDu cells and at 12.5–25-50 nM in SALTO-5 cells. An increasing content of the hypodiploid DNA in the subG1 phase is typical of apoptosis. The treatment with Bortezomib also induced, at all doses tested, a significant increase in A-253 cells for the percentage of cells in the G2/M phase. The increase of the cells in G2/M phase is indicative of the arrest of the cell cycle at the checkpoint to prevent mitosis of cells with damaged DNA (Fig. 2 and Supplementary Fig. S1). The mean result of two independent experiments were reported in Fig. 2. Representative flow cytometry plots in which the effect of Bortezomib on DNA content was compared to that obtained with DMSO in the different cell lines were reported in Supplementary Fig. S1.

To confirm the induction of apoptosis, cells were simultaneously treated with Bortezomib at the highest dose and with the universal caspase inhibitor, Z-VAD-FMK. Administration of this drug was able to significantly reduce the number of cells in the subG1 phase as compared to the single treatment with Bortezomib at the highest dose, thus suggesting the induction of cell death by apoptosis following treatment with Bortezomib in HNC cell lines (Fig. 2 and Supplementary Fig. S1).

### Effect of Bortezomib on the expression of molecules involved in apoptosis in HNC cell lines

**Figure 3:**
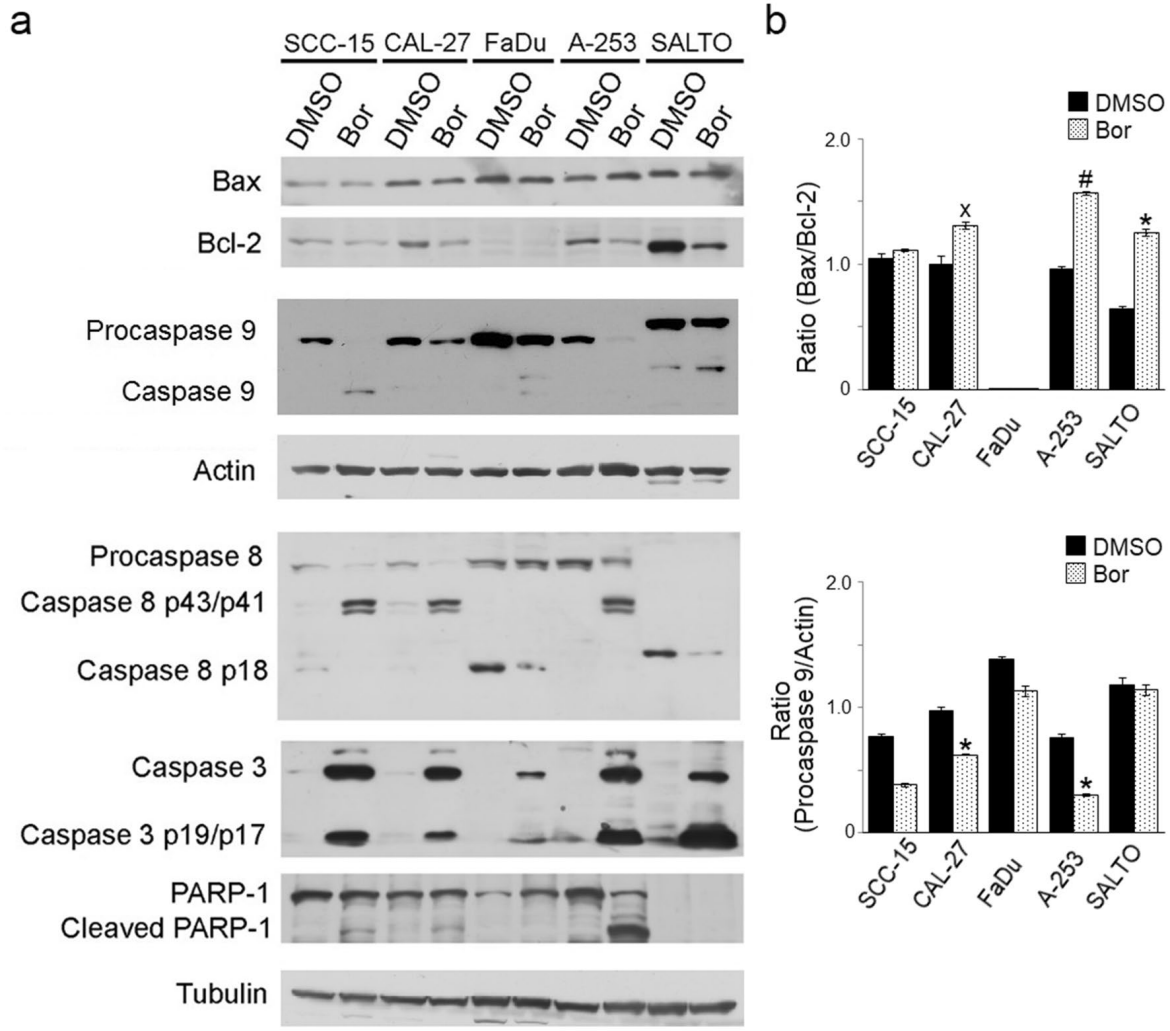
Effect of Bortezomib on the expression of molecules involved in apoptosis in tongue (SCC-15, CAL-27), pharynx (FaDu), and salivary gland (A-253, SALTO-5) cancer cell lines. (**a**) The expression of Bax, Bcl-2, caspases 9, 8, 3, and PARP-1 was evaluated by Western blotting analysis following treatment for 24 or 48 h of HNC cells with Bortezomib (Bor) at a dose of 12.5 (SCC-15, CAL-27) or 25 nM or with DMSO, as reported in Materials and Methods. Actin and tubulin were used as an internal control. (**b**) The densitometric ratios between Bax and Bcl-2, between procaspase 9 and actin, and statistical analysis are reported (˟*p* ≤ 0.05, *p ≤ 0.01, #*p* ≤ 0.001 *vs* DMSO). Data are expressed as the mean ± SD of two independent experiments. Uncropped western blots are reported in Supplementary Informations.

To corroborate that Bortezomib treatment induced apoptosis, the expression of Bax, Bcl-2, procaspases 9/8/3, caspases 9/8/3 and cleavage of poly (ADP-ribose)polymerase-1 (PARP-1) was analyzed by Western blotting after treatment of cells with different concentrations of Bortezomib, as indicated in Fig. 3. Our results showed that Bortezomib treatment significantly increased the Bax/Bcl-2 ratio in CAL-27 (*p* = 0.023), A-253 (*p* = 0.0007) and SALTO-5 (*p* = 0.0015) cells. In SCC-15 and FaDu cells this ratio remained unchanged as compared to control cells. However, the activation of the intrinsic pathway of apoptosis was observed in all cell lines. Indeed, our results showed that Bortezomib treatment was able to induce the release of the active fragment of procaspase-9 in SCC-15, FaDu and SALTO-5 cells and to reduce the expression of procaspase 9 in the other cell lines, thus suggesting the activation of the molecule (CAL-27, *p* = 0.0033; A-253, *p* = 0.0025). In addition, the activation of the extrinsic pathway of apoptosis was observed as well in SCC-15, CAL-27 and A-253 cells, as indicated by the cleavage of the procaspase 8 in the activated fragments p43/41. In the other cell lines, the activation of the extrinsic pathway of programmed cell death was not detected (Fig. 3).

Activated caspases 9/8 can cleave and activate caspase 3, inducing the proteolytic inactivation of PARP-1, which is involved in DNA repair and genomic integrity. Indeed, our results showed that Bortezomib caused in all cell lines the proteolytic cleavage of caspase 3 into the activated fragments p19 and p17, and the proteolytic cleavage of PARP-1 in SCC-15, CAL-27 and A-253 cells (Fig. 3).

### Effects of Bortezomib on the expression and activation of ErbB receptors (EGFR and ErbB2) and pro-survival signaling transduction pathway molecules (ERK, JNK, p38, AKT) in HNC cell lines

The MAP (Mitogen Activated Protein) kinase transduction pathway is triggered by the activation of EGFR and ErbB2/*neu* tyrosine kinase receptors. It has been demonstrated that HNC cell lines overexpress EGFR and ErbB2 receptors, which play a role in their cellular transformation^35^.

**Figure 4:**
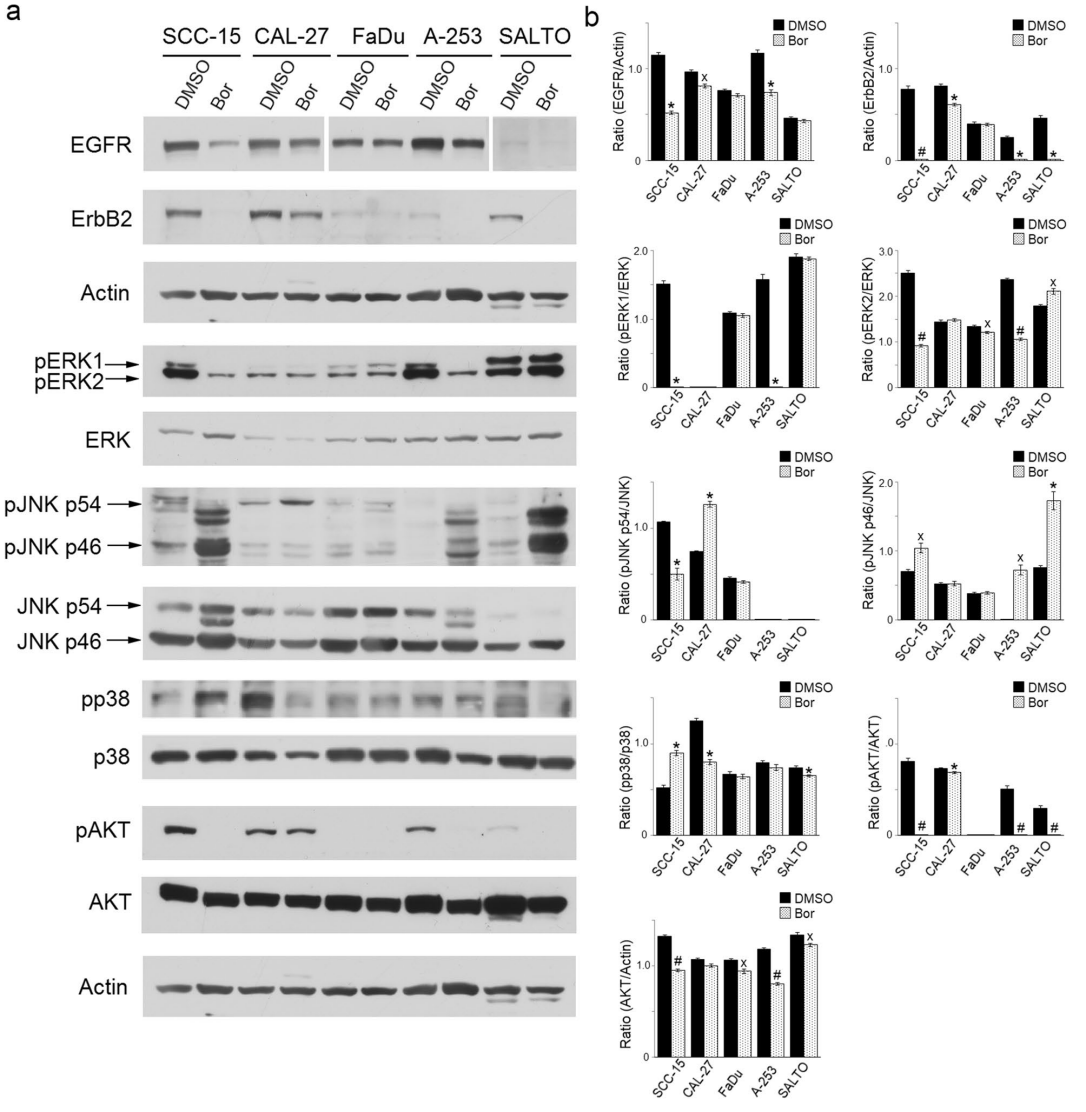
Effect of Bortezomib on the expression and activation of ErbB receptors (EGFR and ErbB2) and signaling transduction pathway molecules. (**a**) Western blotting analysis was performed on HNC cells treated with Bortezomib (Bor) or DMSO for 24 or 48 h, at a dose of 12.5 (SCC-15, CAL-27) or 25 nM, as reported in Materials and Methods. The levels of pERK1, pERK2, pp38, pJNK, as well as pAKT, were compared with that of total ERK, p38, JNK, and AKT proteins, respectively. The intensity of the bands was quantified using the ImageJ 1.53e software after blot scanning of two independent experiments. Actin was used as an internal control. (**b**) The densitometric ratios and statistical analysis are reported (˟*p* ≤ 0.05, *p ≤ 0.01, #*p* ≤ 0.001 *vs* DMSO). Data are expressed as the mean ± SD of two independent experiments. Uncropped western blots are reported in Supplementary Informations.

Our results indicated that Bortezomib significantly decreased the level of EGFR and ErbB2 expression in SCC-15 (*p* = 0.0014, for EGFR; *p* = 0.0006, for ErbB2), CAL-27 (*p* = 0.013, for EGFR; *p* = 0.0018, for ErbB2), A-253 (*p* = 0.0067, for EGFR; *p* = 0.002, for ErbB2) cells as compared to control cells. Only a decreased expression of ErbB2/*neu* (*p* = 0.007) was observed in the murine cell line SALTO-5. In contrast, the expression of these receptors remained unchanged in FaDu cell line (Fig. 4).

In addition, we evaluated the effect of Bortezomib on the expression and phosphorylation of MAP kinases ERK, JNK and p38. Our results showed that Bortezomib inhibited the phosphorylation of ERK1 and ERK2 in SCC-15 (*p* = 0.0027, for pERK1; *p* = 0.0009, for pERK2) and A-253 (*p* = 0.0043, for pERK1; *p* = 0.0003, for pERK2) cells as compared to untreated cells. In addition, Bortezomib treatment decreased the level of phosphorylation of ERK2 in FaDu cells (*p* = 0.016), while it increased it in SALTO-5 cells (*p* = 0.019). Bortezomib did not affect the levels of phosphorylation of ERK1/2 in CAL-27 cells (Fig. 4).

Furthermore, it was observed that Bortezomib decreased the level of phosphorylation of JNK p54 in SCC-15 cells (*p* = 0.006), while increased it in CAL-27 (*p* = 0.0016) and that of JNK p46 in SCC-15 (*p* = 0.022), A-253 (*p* = 0.020) and SALTO-5 cells (*p* = 0.009). Bortezomib decreased the p38 phosphorylation in CAL-27 (*p* = 0.0037), and SALTO-5 (*p* = 0.002) cells, while the p38 phosphorylation was enhanced in SCC-15 cells (*p* = 0.003). Bortezomib did not affect the phosphorylation of p38 in FaDu and A-253 cells (Fig. 4).

Finally, we evaluated whether Bortezomib treatment inhibited the expression and phosphorylation of the pro-survival kinase AKT, which promotes tumor growth^36^. Our results indicated that Bortezomib decreased AKT phosphorylation in SCC-15 (*p* < 0.001), CAL-27 (*p* = 0.0011), A-253 (*p* < 0.001) and SALTO-5 cells (*p* < 0.001) (Fig. 4).

### Effects of Bortezomib on autophagy of HNC cell lines

**Figure 5:**
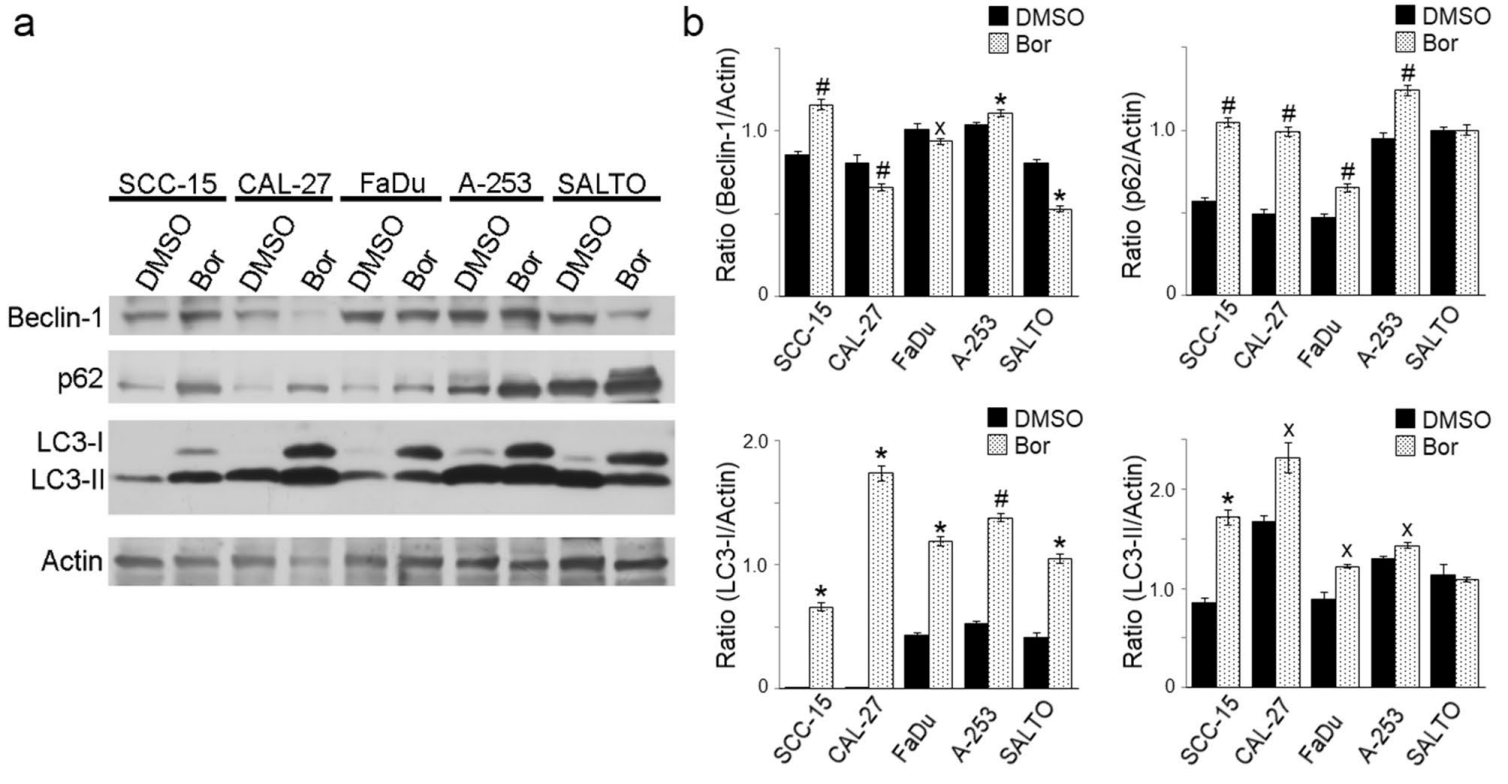
Effect of Bortezomib on autophagy in HNC cells. (**a**) Western blotting analysis was performed on HNC cells treated with Bortezomib (Bor) or DMSO for 24 or 48 h, at the dose of 12.5 (SCC-15, CAL-27) or 25 nM, as reported in Materials and Methods. The intensity of the bands was quantified using the ImageJ 1.53e software after blot scanning of two independent experiments. Actin was used as an internal control. (**b**) The densitometric ratios between Beclin-1 and actin, p62 and actin, LC3-I and actin, LC3-II and actin, and statistical analysis are reported (˟*p* ≤ 0.05, *p ≤ 0.01, #*p* ≤ 0.001 *vs* DMSO). Data are expressed as the mean ± SD of two independent experiments. Uncropped western blots are reported in Supplementary Informations.

To determine the effect of Bortezomib in the induction of autophagy, HNC cell lines were analyzed for the expression of Beclin-1, LC3-I, LC3-II and p62 by Western blotting. Our results showed that Bortezomib significantly increased Beclin-1 expression in SCC-15 (*p* = 0.0007) and A-253 (*p* = 0.0015) cells, while it was decreased in CAL-27 (*p* = 0.0005), FaDu (*p* = 0.011) and SALTO-5 (*p* = 0.0021) cells (Fig. 5). The activation of the autophagic process can be revealed by the conversion of the cytosolic form LC3-I into the membrane-bound form LC3-II, which is localized in the autophagosomes^37,38^. Our results showed that LC3-I and LC3-II were constitutively expressed in DMSO-treated cells. Bortezomib significantly increased the expression of LC3-I (SCC-15, *p* = 0.0011; CAL-27, *p* = 0.0011; FaDu, *p* = 0.0012; A-253, *p* = 0.0008) and LC3-II (SCC-15, *p* = 0.006; CAL-27, *p* = 0.031; FaDu, *p* = 0.021; A-253, *p* = 0.039) in all human cell lines. An increase of only LC3-I expression was observed in SALTO-5 cells (*p* = 0.0018) (Fig. 5). In addition, Bortezomib induced the increase of p62 in SCC-15 (*p* = 0.0007), CAL-27 (*p* = 0.00015), FaDu (*p* = 0.00014), A-253 (*p* = 0.0008) cell lines, while its expression did not change in SALTO-5 cells (Fig. 5). These results suggest that Bortezomib induced accumulation of p62 and LC3-II, which is indicative of a blocked autophagy^39^.

### Validation of proteasome inhibition by Bortezomib in HNC cell lines

**Figure 6:**
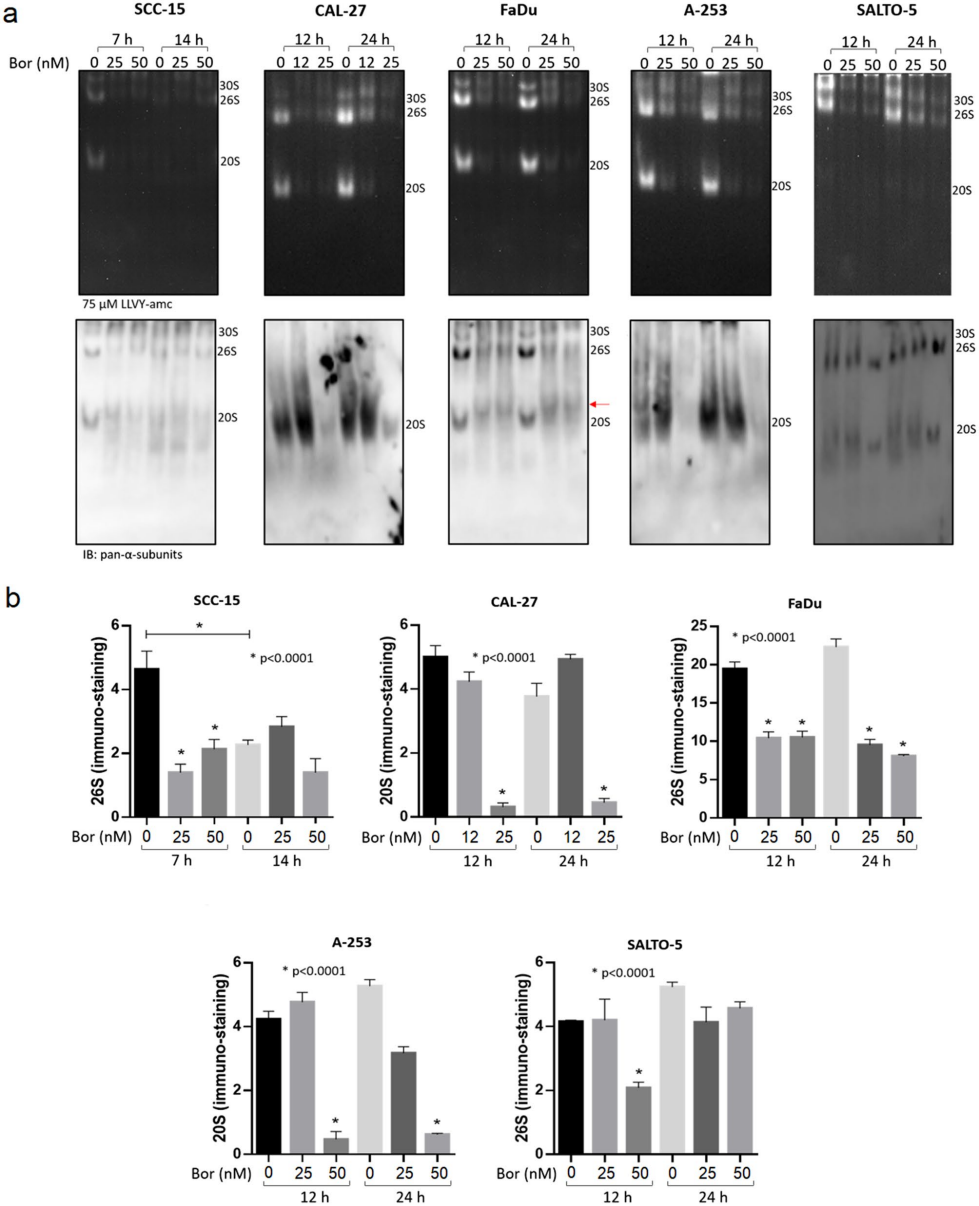
Analysis of structural and functional properties of proteasome particles in the presence of Bortezomib by native-gel electrophoresis coupled with Western blotting. (**a**) Proteasome particles were separated by native-gel electrophoresis and probed with 75 µM LLVY-amc (*upper panel*). The light intensity has a linear correlation with the in gel peptidolytic activity. The identity of the particles has been probed with an anti-pan-α-subunits antibody (*bottom panel*). The red arrow indicates the appearance of non-canonical proteasome assemblies in FaDu cells treated with Bortezomib (Bor) (*bottom panel*). (**b**) Proteasome content was calculated by determining the intensity of the 26S immunostaining for SCC-15, FaDu, SALTO-5 cells and of the 20S for A-253 and CAL-27, that is the most represented species for each cell line assayed. A representative panel of a single experiment is shown. Every assay has been carried out three independent times. Data are presented as mean ± SD (n = 3). One-way ANOVA followed by Tukey’s post-hoc significance test. The reported statistical analysis refers to the ratio between each Bortezomib-related experimental condition *vs* untreated cells at the same time-point.

In order to evaluate the effectiveness of proteasome inhibition in HNC cells by Bortezomib and to determine whether resistance to the drug may have occurred, SCC-15, CAL-27, FaDu, A-253 and SALTO-5 cells were treated with Bortezomib by following a scheme and doses based on results from the IC_50_ values (see Fig. 2). At the indicated time points, Bortezomib- and DMSO-treated cells were harvested and the cytosolic fraction (i.e., where proteasome is mainly represented) was isolated through a non-denaturing lysis procedure. For every cell line, proteasome inhibition and individual particles content was first assayed by native gel electrophoresis^40^ (see Materials and Methods for further details) (Fig. 6).

For all cell lines, a marked inhibition of the activity of all three main assemblies existing in the cell cytosol (*i.e.*, the uncapped 20S, the doubly 19S:20S:19S and single-capped 19S:20S, respectively) was observed in the presence of Bortezomib (Fig. 6a). With respect to control cells, SCC-15, CAL-27, and SALTO-5 cells displayed the greatest extent of proteasome inhibition (> 50%) in the presence of the lowest Bortezomib concentration (25 nM in SCC-15 and SALTO-5, 12.5 nM in CAL-27) at the first time-point (7 h in SCC-15 and 12 h in SALTO-5 and CAL-27) of stimulation (Fig. 6a, *upper panel*; Supplementary Fig. S2). Conversely, in the case of A-253 cells, a > 50% extent of proteasome inhibition at 12 h of stimulation was achieved only at 50 nM Bortezomib. Interestingly, FaDu cells showed a less marked inhibition of proteasome activity as compared to the other cell lines. The extent of inhibition was roughly 50% in the presence of either 25 or 50 nM Bortezomib at 12 h and slightly lower at 24 h (Fig. 6a, *upper panel*; Supplementary Fig. S2). A modest rescue of proteasome activity was further observed in SALTO-5 cells stimulated with 25 and 50 nM Bortezomib for 24 h, as compared to the effect induced by the same Bortezomib concentrations after 12 h of stimulation (Fig. 6a, *upper panel*; Supplementary Fig. S2). In the case of SCC-15 cells, an unexpected finding was observed. The overall proteolytic activity was found to decrease in untreated cells over time (14 h *vs* 7 h) (Fig. 6a *upper panel*; Supplementary Fig. S2).

In accordance with a general inhibition of proteasome activity, the poly-ubiquitinated proteins, (*i.e.*, the natural substrates of capped proteasome assemblies), which were assayed by denaturing and reducing Western blotting, turned out to be significantly increased in the presence of Bortezomib under all experimental conditions (Supplementary Fig. S3).

To further validate their identity, proteasome particles were then transferred to a nitrocellulose filter and probed with an antibody which recognizes all catalytic assemblies of proteasome (*i.e.*, 30S, 26S, and 20S), since it is raised against a peptide covering residues shared by the α1-7 subunits of 20S proteasome, with the exception of α4 (hereafter referred to as pan-α-subunits). By probing the filters some unexpected findings were observed (Fig. 6a, *bottom panel*, and Fig. 6b). First of all, proteasome particles immuno-staining was significantly impaired over the whole time-interval (*i*) in FaDu cells under all tested conditions, (*ii*) in SCC-15 cells stimulated with 25 and 50 nM Bortezomib for 7 h and (*iii*) in A-253 and CAL-27 cells stimulated with the highest Bortezomib concentrations (*i.e.*, 50 and 25 nM, respectively) (Fig. 6a, *bottom panel*, and Fig. 6b); (*iv*) in SALTO-5 cells treated with 50 nM Bortezomib over 12 h. Remarkably, regardless the dose and time of Bortezomib incubation, FaDu cells treated with Bortezomib showed the appearance of non-canonical proteasome assemblies with an apparent mass/charge ratio very similar to that reported by other authors in a similar experimental condition^41^ (Fig. 6a, *bottom panel*, red arrow). It is worth pointing out that SCC-15 cells displayed a decrease in overall proteasome content also in control cells harvested after 14 h (with respect to control cells harvested after 7 h), thus confirming the observed loss of proteasome activity (Fig. 6). Conversely, SALTO-5 cells appeared to be somewhat resistant to this phenomenon, since this cell line did not display any significant decrease of proteasome particles content (Fig. 6a, *bottom panel*, and Fig. 6b) with the exception of cells treated with 50 nM for 12 h.

In addition, A-253 and CAL-27 cells displayed a very high 20S/capped assemblies (30S + 26S) ratio, even though the pattern of activity (Fig. 6a, *bottom panel*, and Fig. 6b) was comparable to that of other cell lines analyzed which showed a more balanced 20S/capped assemblies ratio.

To better address these findings, the same crude cell extracts, run by native gel electrophoresis, were analyzed by denaturing, and reducing Western blotting. Interestingly, the content of free α7 and Rpt5 subunits, which are representative of the 20S and 19S particles, respectively, ranged from unchanged or even increased under the investigated experimental conditions (Supplementary Fig. S3).

**Figure 7:**
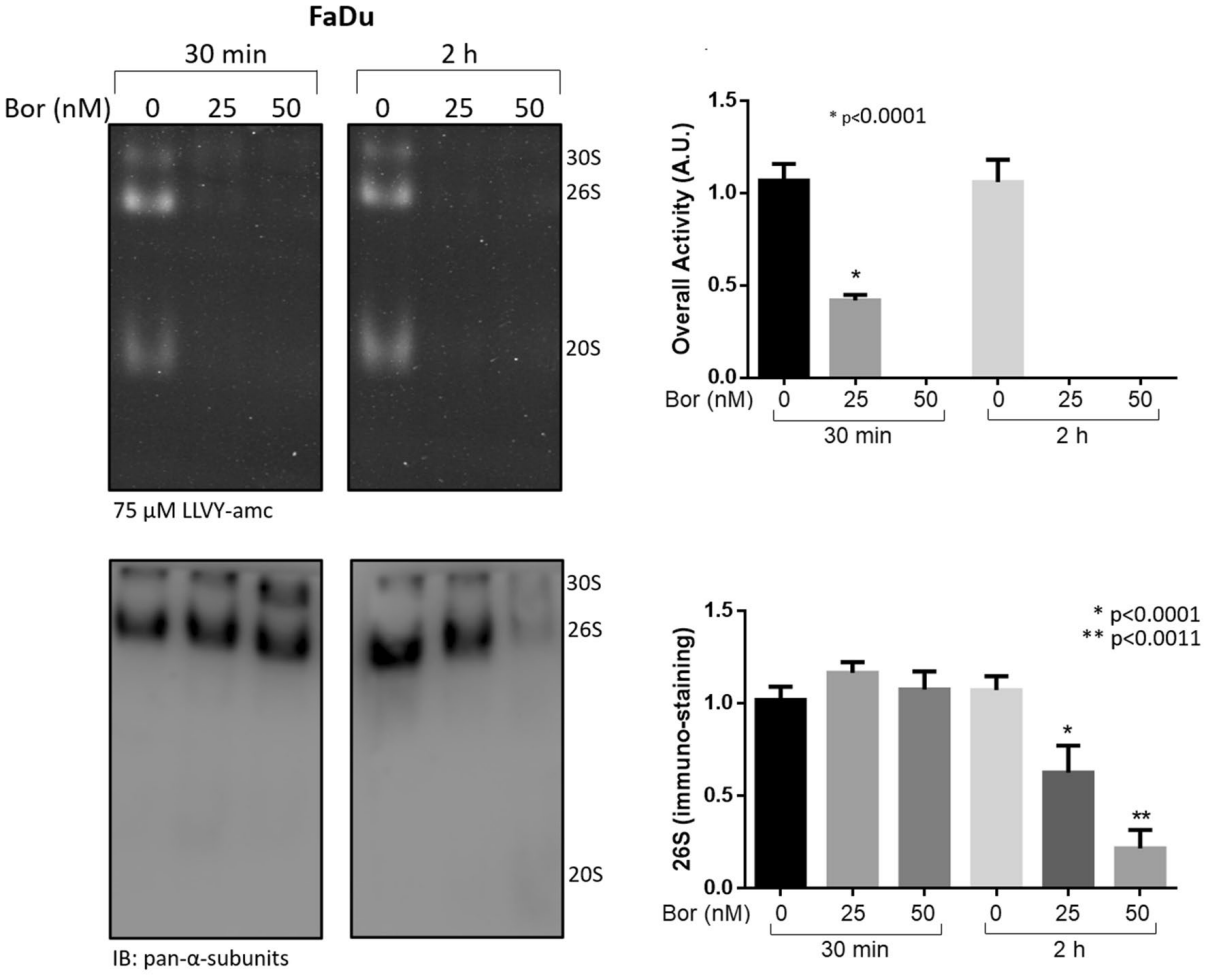
Analysis of proteasome particles at early times after Bortezomib stimulation in FaDu cell line. FaDu cells were treated with 25 and 50 nM Bortezomib (Bor) for 30 min and 2 h and analyzed as described previously by native-gel electrophoresis followed by Western Blotting (*left panel*). Densitometric analysis of proteasome particles activity was determined by in-gel peptidolytic activity and that of proteasome immunostaining by Western Blotting (*right panel*). A representative panel of a single experiment is shown. Every assay has been carried out three independent times. Data are presented as mean ± SD (n = 3). One-way ANOVA followed by Tukey’s post-hoc significance test. The reported statistical analysis refers to the ratio between each Bortezomib-related experimental condition *vs* untreated cells at the same time-point.

To further verify the observed phenomenon, FaDu cells, which displayed the apparent highest extent of proteasome loss, were further stimulated with 25 and 50 nM Bortezomib for 30 min and 2 h (Fig. 7). Proteasome activity was almost null as early as after 30 min of stimulation in the presence of 50 nM Bortezomib, whilst a residual activity was observed in the presence of 25 nM Bortezomib (Fig. 7, *left panel*). After 2 h of stimulation, the proteolytic activity was undetectable in the presence of all Bortezomib concentrations. Interestingly, by probing the filter with the anti-pan-α subunits antibody, it was observed that the proteasome assemblies were unaffected by Bortezomib after 30 min of stimulation (Fig. 7, *left panel*). Conversely, after 2 h of Bortezomib treatment, a very robust loss of proteasome assemblies was observed in the presence of 50 nM Bortezomib, whereas residual proteasome assemblies were detected in the presence of 25 nM Bortezomib (Fig. 7, *right panel*).

### Toxicological evaluation of Bortezomib treatment by histological analysis

Hematoxylin and eosin staining was performed on tissue sections from multiple organs (liver, lung, kidney, spleen, heart) collected from BALB-*neu*T mice treated i.p. with Bortezomib for 4 weeks. Our results showed no cytological and architectural alterations in any of the organ examined. In particular, hematoxylin and eosin staining showed a well-preserved liver parenchyma with a normal portal triad; no alterations in both renal glomeruli and tubules; a well-preserved lung parenchyma; no signal of hypertrophy and inflammation in the heart; a normal architecture of the spleen (Supplementary Fig. S4).

### Bortezomib inhibited the in vivo growth of SALTO-5 cells transplanted in BALB-*neu*T mice

**Figure 8:**
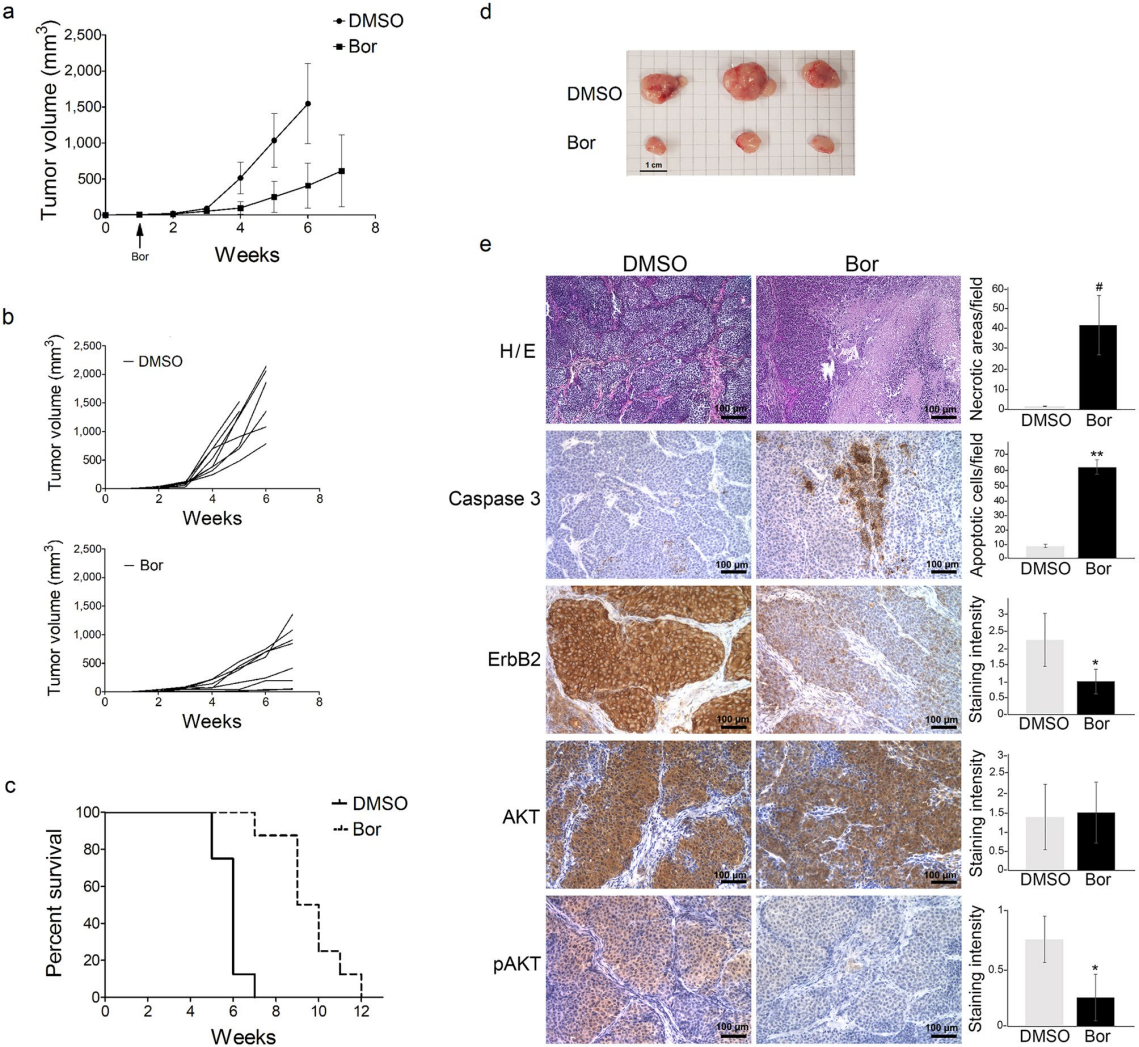
Bortezomib reduced tumor growth and increased the survival in BALB-*neu*T mice that were subcutaneously inoculated with SALTO-5 cells. (**a**) Differences in mean tumor volumes ± SD between BALB-*neu*T mice treated with Bortezomib (Bor) or PBS + DMSO. (**b**) Curves representing tumor growth in single mice treated with Bortezomib (Bor) or PBS + DMSO. (**c**) Differences in the mean survival duration of BALB-*neu*T mice treated with Bortezomib (Bor) or PBS + DMSO. (**d**) Representative photographs of explanted tumor masses after 5 weeks. Scale bar corresponds to 1 cm. (**e**) Histology and immunohistochemical analysis of tumors from Bortezomib-treated mice (Bor) or control-treated mice (PBS + DMSO). Tumors from three different mice for each group were stained using hematoxylin and eosin (H/E). Necrotic areas were quantified by ImageJ 1.53e software on 10 representative microscopic fields. Percentage average of necrotic areas are reported in the graph. IHC was performed to analyze the expression of cleaved caspase 3, ErbB2, AKT and phospho-AKT (pAKT) in tumor samples. Tumor tissues from three mice in each group were analyzed and representative images were reported. The number of cleaved caspase 3-positive cells (apoptotic cells) within the tumors was evaluated on 10 representative microscopic fields and the results are shown in the adjacent bar graph. Intensity of ErbB2, AKT and pAKT expression within the tumors was semiquantitative evaluated as described in Materials and Methods, and the results are shown in the adjacent bar graph (#: *p* = 0.0015; *: *p* = 0.04; **: *p* = 0.0001). Scale bars correspond to 100 µm.

Treatment with Bortezomib significantly affected tumor growth in vivo. BALB-*neu*T mice were subcutaneously injected in the right flank with SALTO-5 cells and intraperitoneal (i.p.) treated with Bortezomib or PBS + DMSO. Tumor volumes in the Bortezomib-treated mice were significantly less than those in the control-treated mice after 3 weeks (53.1 *vs* 90.5 mm^3^; *p* = 0.030) (Fig. 8a,b). This significant difference was maintained after 4 weeks (97.4 *vs* 513.5 mm^3^; *p* = 0.0002) and until the 5th week, when two control-treated mice were sacrificed due to the excessive size of the tumor (251.6 *vs* 1035 mm^3^; *p* = 0.0001) (Fig. 8d). The remaining control-treated mice were sacrificed at 6 (5 mice) and 7 (1 mouse) weeks (Fig. 8a,b). At this stage (7 weeks after tumor challenge) only one Bortezomib-treated mouse was sacrificed due to excessive size of the tumor. In contrast, the remaining Bortezomib-treated mice were sacrificed at week 9 (3 mice), 10 (2 mice), 11 (1 mouse) and 12 (1 mouse) (Fig. 8a,b). The mean survival significantly increased for the mice treated with Bortezomib, as compared to the control-treated mice (9.5 *vs* 6 weeks, Bortezomib-treated mice *vs* control-treated mice; *p* = 0.0001) (Fig. 8c), indicating that the risk of SALTO-5 cell growth in control-treated mice was 22.57 times greater than that of mice treated with Bortezomib [95% Hazard Ratio Confidence Limits: lower, 4.524; upper, 112.6; log-rank test (Mantel-Cox)]. Our results demonstrated that treatment with Bortezomib interfered with the in vivo tumor growth of transplanted salivary gland cancer cells SALTO-5.

### Histological analysis of tumors from mice treated with Bortezomib by Optical Microscopy

Histological examination of tumors from control mice (PBS + DMSO, n = 3) showed the presence of solid tumors with the histological features of the adenocarcinomas; very small areas of cellular alterations and/or necrosis were observed. In contrast, histological examination of tumors from Bortezomib-treated mice (Bor, n = 3) showed extensive necrosis as compared to control mice (41.5 ± 14.5 *vs* 1.8 ± 0.2 percentage of necrotic areas/microscopic field, *p* = 0.0015) (Fig. 8e).

The presence of apoptotic cells was evaluated by the expression of cleaved caspase 3 in cancer cells employing immunohistochemical analysis (IHC) (Fig. 8e). The number of apoptotic cells within the tumors from Bortezomib-treated mice (62.8 ± 5.1) was higher than that from control-treated mice (8.4 ± 1.1) (*p* = 0.0001).

The expression of ErbB2 on tumor cells *in vivo* was evaluated by IHC as well (Fig. 8e). Tumors from Bortezomib-treated mice showed a significantly lower expression of ErbB2 than that from control-treated mice (1.0 ± 0.4 *vs* 2.3 ± 0.8 staining intensity, *p* = 0.04). In addition, AKT phosphorylation was significantly decreased in tumors from mice treated with Bortezomib, as compared to those treated with DMSO (0.3 ± 0.2 *vs* 0.8 ± 0.2 staining intensity, *p* = 0.04). On the other hand, the same treatment did not affect AKT expression in tumors (Fig. 8e).

**Figure 9:**
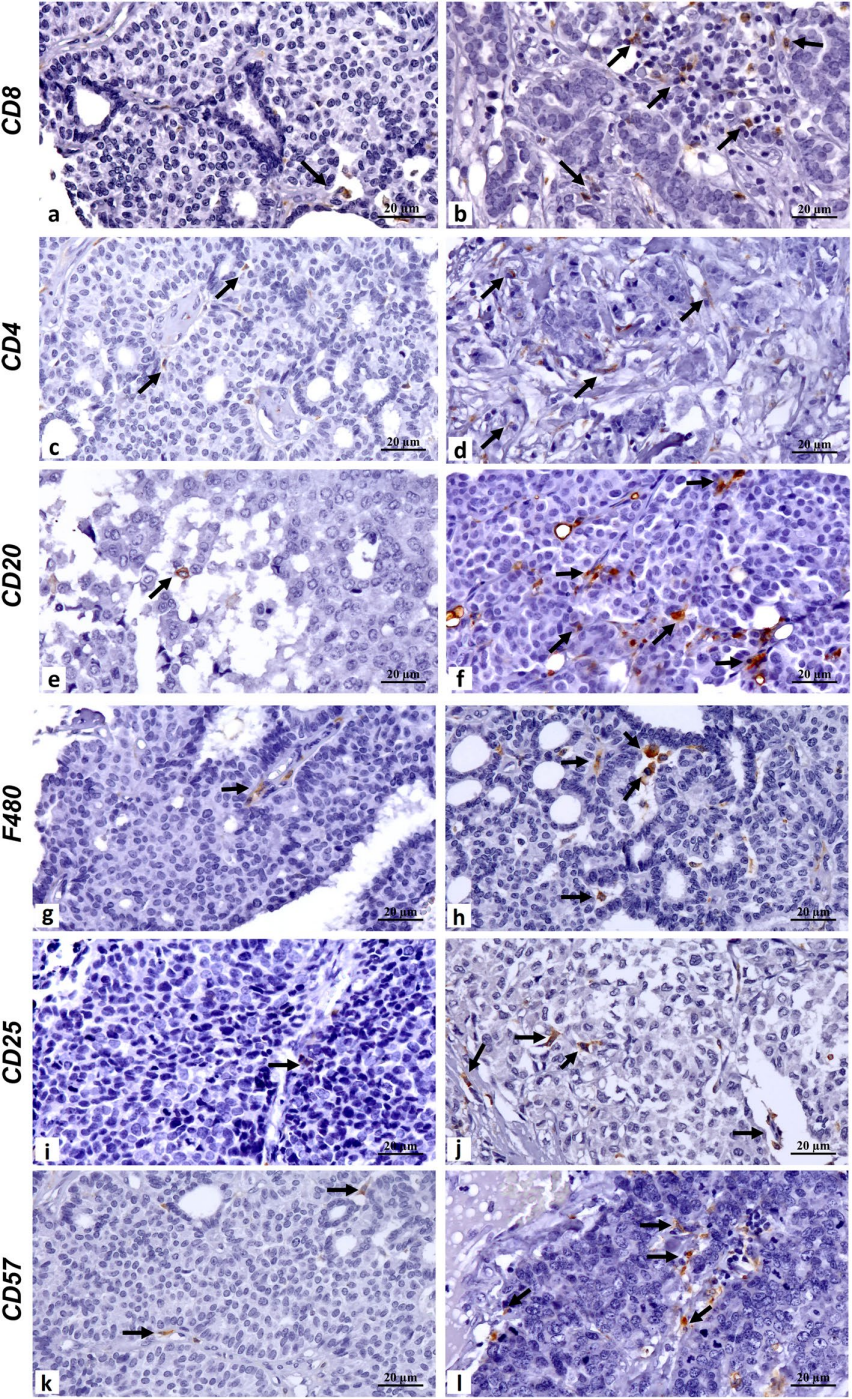
Immunohistochemical evaluation of CD8^+^, CD4^+^, CD20^+^, F480^+^, CD25^+^ and CD57^+^ immune cells. IHC analysis of tumors from Bortezomib-treated mice (Bor) or control-treated mice (PBS + DMSO). Tumor tissues from three mice in each group were analyzed and representative images were reported. (**a**) Image shows rare CD8^+^ lymphocytes (arrow) in a control-treated tumor. (**b**) Numerous intratumoral CD8^+^ lymphocytes (arrows) in a tumor treated with Bortezomib. (**c**) CD4^+^ immunohistochemical stain displays rare intratumoral lymphocytes (arrows) in a control-treated sample. (**d**) A Bortezomib-treated tumor sample characterized by several CD4^+^ lymphocytes (arrows). (**e**) A CD20^+^ intratumoral cell (arrow) in a control-treated tumor. (**f**) Moderate CD20^+^ inflammatory infiltrate (arrows) in a tumor treated with Bortezomib. (**g**) Image shows rare macrophages (arrow) in a control-treated tumor. (**h**) Numerous large macrophages (arrows) in a tumor treated with Bortezomib. (**i**) CD25^+^ immunohistochemical stain displays rare intratumoral lymphocytes (arrow) in a control-treated sample. (**j**) Moderate CD25^+^ immune infiltrate (arrows) in a tumor treated with Bortezomib. (**k**) A control-treated tumor characterized by rare CD57^+^ cells (arrows). (**l**) A Bortezomib-treated tumor sample characterized by several NK (CD57^+^) cells (arrows). Scale bars correspond to 20 µm.

Moreover, we investigated the effects of Bortezomib treatment on the number of tumor-infiltrating immune cells. The presence and/or modifications of the immune infiltrate was analyzed by IHC analysis by evaluating the number of CD4^+^, CD8^+^, CD57^+^, CD25^+^, F480^+^ and CD20^+^ cells. In addition, tumor vessels density and expression of some proteasome structural subunits (PSMA4, PSMD4, PSME1) and ubiquitin were evaluated. Our results demonstrated that Bortezomib treatment deeply affected the number of tumor-infiltrating immune cells. Indeed, a significant increase in the number of helper T lymphocytes (CD4^+^), cytotoxic T lymphocytes (CD8^+^), B lymphocytes (CD20^+^) and macrophages (F480^+^) were observed in the Bortezomib-treated group as compared to the control-treated mice (Fig. 9 and Table 2). In addition, Bortezomib-treated tumors showed a significant increase in NK cells (CD57^+^) and CD25^+^ immunoregulatory T cells (Fig. 9 and Table 2). These results suggest that Bortezomib can potentiate the recruitment of tumor-infiltrating immune cells. Moreover, a significant reduction in tumor vessels density from Bortezomib-treated mice, as compared to those from control-treated mice was observed (Supplementary Fig. S5 and Table 2).

**Table 2:** Immunohistochemical evaluation of markers for tumor infiltrating cells and molecules involved in the proteasome activity.

	PBS + DMSO (mean ± SD)	Bortezomib (mean ± SD)	*p* Value*
**Tumor infiltrating cells**^a^			
CD8	0.27 ± 0.46	3.13 ± 1.30	< 0.0001
CD4	0.33 ± 0.49	1.40 ± 0.74	< 0.0001
CD20	0.13 ± 0.35	1.60 ± 0.83	< 0.0001
F480	0.13 ± 0.35	2.27 ± 0.88	< 0.0001
CD25	0.27 ± 0.46	2.00 ± 1.19	< 0.0001
CD57	0.67 ± 0.72	3.53 ± 0.99	< 0.0001
**Vessels marker**^**b**^			
CD34	2.60 ± 1.81	1.27 ± 1.03	0.02
**Proteasome proteins**^**c**^			
PSMA4	2.40 ± 0.83	3.47 ± 0.74	0.001
PSMD4	3.33 ± 0.98	4.87 ± 0.92	0.0001
PSME1	4.53 ± 0.99	3.00 ± 0.93	0.0002
Ubiquitin	4.27 ± 0.96	4.20 ± 1.15	0.864

*Two-tailed Student’s T test.

^a^Positive cell count/HPF averaging 5 representative microscopic fields.

^b^Positive vessel count/HPF averaging 5 representative microscopic fields.

^c^A combined scoring system was used for the IHC evaluation. Specifically, the total score (0–6) was obtained by adding the score associated to the number of positive cells and the score related to the signal intensity. The score associated to the number of positive cells was defined as follow: 0 (1 ≤ Positive cells/HPF), 1 (2 ≤ x ≤ 10 Positive cells/HPF), 2 (11 ≤ x ≤ 20 Positive cells/HPF), 3 (≥ 21 Positive cells/HPF). The score related to the signal intensity was defined as follow: 0 (absent/very low Intensity/HPF), 1 (low Intensity/HPF), 2 (moderate Intensity/HPF), 3 (high Intensity/HPF).

To investigate whether Bortezomib affected T cells activation and IFN-γ production, intratumoral and spleens (SPL) lymphocytes from Bortezomib- or PBS + DMSO-treated mice carrying transplanted (SALTO-5) cells were collected and analyzed by flow cytometry. T cells CD69 expression was employed to measure T cells activation. Tumor infiltrating CD4^+^ and CD8^+^ lymphocytes (TIL) were highly activated and CD8^+^ lymphocytes were particularly prone to IFN-γ production both in Bortezomib- and in DMSO-treated mice (frequency of CD4^+^CD69^+^: Bor 35.73 *vs* DMSO 33.37, *p* = 0.863; frequency of CD8^+^CD69^+^: Bor 36.80 *vs* DMSO 35.56, *p* = 0.867; frequency of CD4^+^IFN-γ^+^: Bor 5.66 *vs* DMSO 5.20, *p* = 0.839; frequency of CD8^+^IFN-γ^+^: Bor 13.45 *vs* DMSO 16.56, *p* = 0.686) (Supplementary Fig. S6). Splenic CD4^+^ and CD8^+^ lymphocytes did not show high activation and IFN-γ production (frequency of CD4^+^CD69^+^: Bor 7.68 *vs* DMSO 6.78, *p* = 0.667; frequency of CD4^+^IFN-γ^+^: Bor 5.00 *vs* DMSO 6.88, *p* = 0.813; frequency of CD8^+^CD69^+^: Bor 1.06 *vs* DMSO 1.01, *p* = 0.845; frequency of CD8^+^IFN-γ^+^: Bor 4.76 *vs* DMSO 3.17, *p* = 0.316) (Supplementary Fig. S6). However, both CD69 expression and IFN-γ production of intratumoral and spleens lymphocytes did not significantly differ between Bortezomib- and DMSO-treated mice (*p* > 0.05). No difference was observed for CD69 and IFN-γ expression in NK cells (CD3^-^CD49b^+^) (TIL: frequency of CD3^-^CD49b^+^CD69^+^: Bor 4.75 *vs* DMSO 5.93, *p* = 0.671; frequency of CD3^-^CD49b^+^IFN-γ^+^: Bor 14.85 *vs* DMSO 19.30, *p* = 0.344; SPL: frequency of CD3^-^CD49b^+^CD69^+^: Bor 8.50 *vs* DMSO 6.96, *p* = 0.269; frequency of CD3^-^CD49b^+^IFN-γ^+^: Bor 14.03 *vs* DMSO 10.030, *p* = 0.396) (Supplementary Fig. S6).

**Figure 10:**
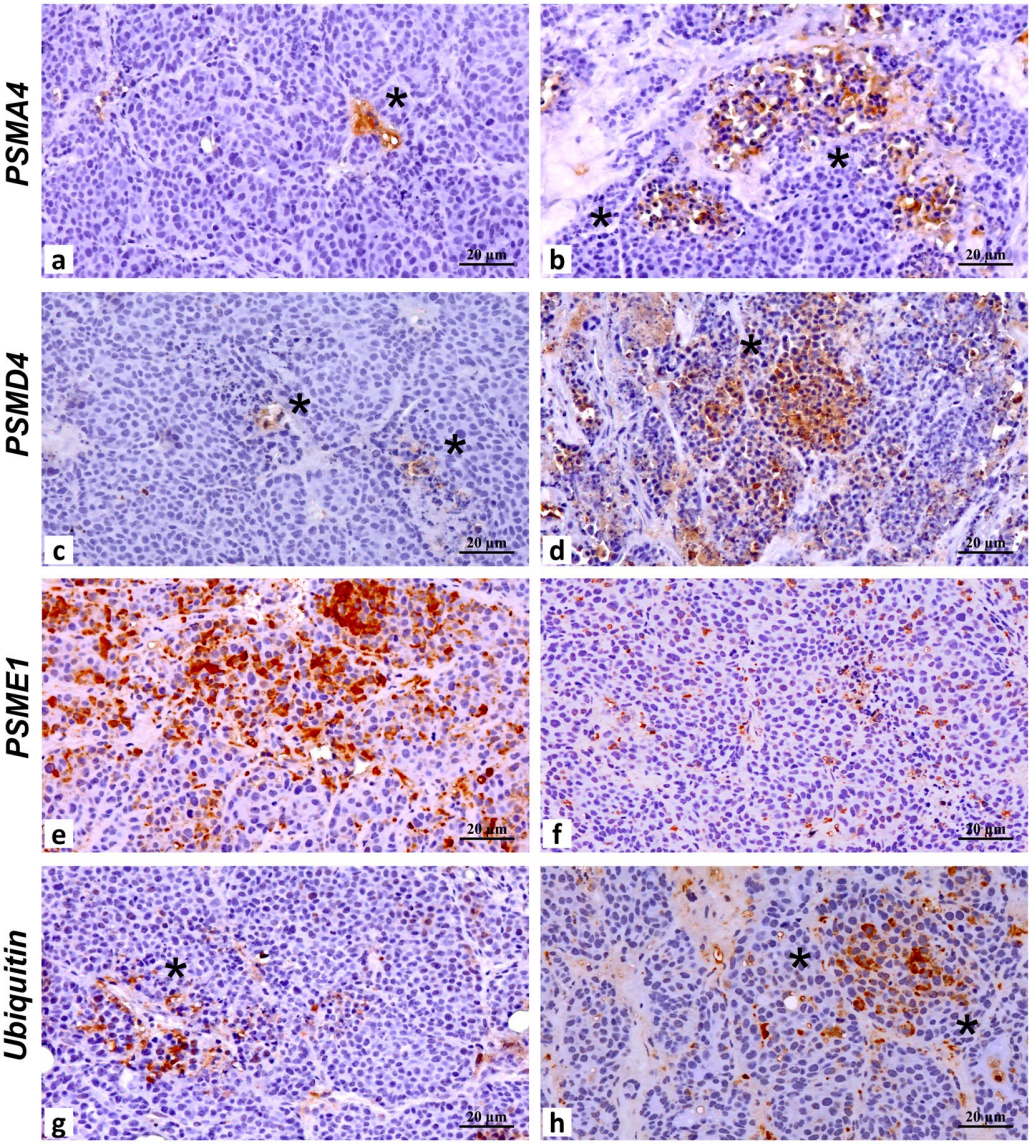
Effect of Bortezomib on proteasome subunits expression in Bortezomib-treated mice (Bor) or control-treated mice (PBS + DMSO) by IHC analysis. Tumor tissues from three mice in each group were analyzed and representative images were reported. (**a**) Image shows modest and isolated PSMA4^+^ cancer cells (asterisk) in a control-treated tumor. (**b**) Numerous and widely distributed PSMA4^+^ cancer cells (asterisks) in a tumor treated with Bortezomib. (**c**) PSMD4 immunohistochemical stain displays isolated clusters of positive cancer cells (asterisks) in a control-treated sample. (**d**) Image shows a tumor area with high expression of PSMD4 (asterisk) after the treatment with Bortezomib. (**e**) A control-treated tumor characterized by numerous PSME1^+^ large cancer cells. (**f**) Isolate PSME1^+^ cancer cells in a tumor treated with Bortezomib. (**g**) Cluster of ubiquitin-positive cancer cells (asterisk) in a control-treated tumor. (**h**) Ubiquitin stain reveals a cluster of positive cancer cells in a Bortezomib-treated tumor. Scale bars correspond to 20 µm.

Proteasome structural subunits, both PSMA4 (e.g., the 20S α3 subunit) and PSMD4 (e.g., the 19S Rpn10 subunit) were faintly detectable in DMSO-treated tumors, with the exception of some isolated foci that displayed stronger immunoreactivity. However, the immunostaining of these subunits turned out to be significantly stronger (*p* < 0.001 for PSMA4, and *p* < 0.0001 for PSMD4) in Bortezomib- than in DMSO-treated tumors (Fig. 10 and Table 2). Strikingly, immunostaining of PSME1 was markedly reduced in Bortezomib- *vs* DMSO-treated tumors (*p* < 0.0002) (Fig. 10 and Table 2). PSME1 encodes for the α subunit of the PA28 complex, a regulatory particle alternative to the canonical 19S, whose expression is often transcriptionally upregulated upon delivery of stressors, at least in vitro. Nevertheless, with respect to DMSO-treated tumors, ubiquitin immunostaining was unaltered between the two experimental groups (Fig. 10 and Table 2).

### Ultrastructural analysis of SALTO-5 cells in vitro treated with Bortezomib

Ultrastructural analysis was performed by transmission electron microscopy on SALTO-5 cells treated with Bortezomib or DMSO at the concentration of 25 nM for 24 h. DMSO-treated cells showed heterogeneous forms with a predominance of round over stretched cells. The nuclei appeared large, mainly formed by euchromatin with low dense heterochromatin in the periphery. In the cytoplasm several mitochondria and cisterns of rough endoplasmic reticulum were detected, with few vacuoles (Supplementary Fig. S7a,b). Conversely, Bortezomib-treated cells showed mainly necrotic features (Supplementary Fig. S7c) with cytoplasm swelling and the presence of numerous cytoplasmic vacuoles surrounded by a single or double membrane, the latter being probably of autophagic origin (Supplementary Fig. S7d). Apoptotic cells were also visible (Supplementary Fig. S7e).

## Discussion

The use of new therapeutic agents is being evaluated in HNC^5–8^. Among the approaches currently employed in clinical trials, the delivery of UPS inhibitors, mainly those targeting proteasome catalytic activity, is gaining considerable interest. The purpose of proteasome inhibitors is to block the turnover of the intracellular proteome, bringing about the formation of a toxic environment and the subsequent need to activate apoptotic processes^42,43^. In this regard, Bortezomib is a reversible inhibitor of the chymotrypsin-like activity of the 20S proteasome^14,20,21^ and it has been reported to possess potent anticancer activity, both in vitro and *in vivo*^23–26,44,45^.

Preclinical studies have shown that Bortezomib, when used alone, inhibited cell growth, induced apoptosis, autophagy, and modulation of individual signaling transduction pathways in HNSCC (Head and neck squamous cell carcinoma) cells *in vitro*^46–56^. To our knowledge, this is the first report that analyzes in a comprehensive and detailed approach, the broaden cellular effects of Bortezomib, thus providing molecular clues to join together the proteasome inhibition with the activation of apoptosis, autophagy, as well as the modulation of cell survival signaling transduction pathways involved in cellular transformation of HNC cell lines, that is a concern which no previous single study has been dealing with. Bortezomib was previously shown to activate apoptosis by modulating the expression of apoptotic proteins, activation of caspases, cleavage of PARP-1 protein and hypodiploidia and phosphatidylserine externalization in HNSCC cell lines^46,48–52,56,57^^.^ Bortezomib was shown to stimulate autophagy in HNSCC cells in two studies showing autophagosomes formation, upregulation of LC3-I, -II, Beclin-1 and the JNK-dependent phosphorylation of Bcl-2 after Bortezomib treatment^58,59^. It was also suggested that autophagy attenuated the Bortezomib cytotoxicity^59^. Bortezomib was able to modulate expression and activation of signaling pathways in HNSCC, by inhibiting AKT activation and mTOR^46,60^, and by upregulating STAT3^55^ in HNSCC cell lines. Only two studies showed that Bortezomib affects proteasome activity by inducing the accumulation of ubiquitylated proteins in larynx (UM-SCC-11A, -11B)^56^ and mouth floor (SCC1) cell lines^59^. It is worth pointing out that the majority of the studies investigated the effect of Bortezomib on the modulation of individual biological pathways being restricted to SCC cell lines arising in head and neck. In our study the in vitro and in vivo effects of Bortezomib were for the first time analyzed in a salivary gland adenocarcinoma experimental model. Salivary gland carcinomas represent 6–8% of HNC, with heterogeneous morphologies and clinical outcomes^3^. The mainstream therapy for these types of cancer, when feasible, is the surgery followed by radiation therapy. Chemotherapy regimens have demonstrated controversial clinical outcomes with low responses for advanced or metastatic malignant tumors, so that new targeted therapies are under evaluation^61–63^. Bortezomib has been evaluated for the treatment of only adenoid cystic carcinoma in combination with doxorubicin in a phase II trial, leading to no complete or partial responses, but only stabilization of disease in patients^32^.

Our findings about apoptosis activation by Bortezomib corroborated previous results employing HNSCC cell lines and showed for the first time Bortezomib-mediated apoptosis in a salivary gland adenocarcinoma cell line (A-253). It is worth remarking that only the A-253 cell line showed a G2/M arrest simultaneous to apoptosis, thus indicating a different response to the drug. This cell cycle pattern after Bortezomib treatment was previously reported in tumor cell lines from larynx cancer^56^, colorectal cancer^64^, non-small cell lung carcinoma^65^, prostate cancer^43^, Ewing's sarcoma^66^, malignant mesothelioma and breast cancer^67^.

In addition, our results indicated that Bortezomib mediated biological effect in a cell line specific-dependent modality. We found a decreased expression of the EGFR and ErbB2 receptors only in tongue, salivary gland but not pharynx cancer cell lines. EGFR and ErbB2 are often over-expressed in HNC cells^35,68^ and are frequently prone to heterodimerization that confers tumor growth advantage^69,70^. Bortezomib inhibited the phosphorylation of ERK1 and/or ERK2 in FaDu, SCC-15 and A-253 cells, while the modulation of the p38 activation induced by the drug was cell lines dependent. Bortezomib induced an increase in the phosphorylated form of JNK p54 and/or p46 in CAL-27, SCC-15, and A-253 cells but not in FaDu cells. This finding corroborated other studies in which it has been shown that Bortezomib induced apoptosis by activating p38 and/or JNK kinase in several types of cancer^71–76^. Our finding extends this observation to the salivary gland adenocarcinoma cell line. In addition, other studies have shown that the activation of JNK kinase is necessary for the activation of death by autophagy in HNSCC cell lines^47,58^. Accordingly, our results showed that Bortezomib induced autophagy in human HNSCC cells, but the process was then blocked, as showed by the increase of p62^39^. The same effect was observed for the first time in the salivary gland adenocarcinoma cell line. The block of the autophagic flux by Bortezomib was reported in ovarian cancer cells, hepatocellular carcinoma cells and endometrial cancer cells^77^, breast cancer cells^78^, and B-Raf-mutated melanoma cells^79^.

Furthermore, the treatment with Bortezomib inhibited AKT phosphorylation and activation, which triggers a cell survival signal^80^, in both the tongue squamous carcinoma cell lines (SCC-15, CAL-27) and in the salivary gland adenocarcinoma cell line (A-253). The inhibition of AKT phosphorylation by Bortezomib is a key molecular event for Bortezomib-mediated apoptosis in HNC^46,60^ and non-small cell lung cancer cells^76^. However, our findings showed that Bortezomib had no effect on AKT expression and phosphorylation on the FaDu pharynx cell line.

Since we observed different responses on the modulation of the signaling pathway molecules in HNC cell lines, we evaluated whether these differences were dependent on a different sensibility of cells to Bortezomib-induced inhibition of proteasome activity. The analysis of the structural and functional effects of Bortezomib on the proteasome assemblies in HNC cell lines is a further novelty of our study. Indeed, only two studies showed that Bortezomib affects proteasome activity by inducing the accumulation of ubiquitylated proteins in larynx^56^ and mouth floor cells^59^. We found that the different responses observed upon Bortezomib treatment in the HNC cell lines could be due to a different extent of proteasome inhibition. Indeed, the pharyngeal carcinoma cell line (FaDu), which is found to be the most resistant to the action of Bortezomib, was that displaying the lowest extent of proteasome inhibition after 12 h and 24 h of stimulation, regardless the Bortezomib concentration administered. It is important to recall that, as highlighted above, Bortezomib was not able to induce modulation of EGFR, ErbB2, JNK, p38 as well AKT proteins in FaDu cells. The ineffectiveness of Bortezomib in modulating these signal transduction pathways may thus parallel the low efficacy of Bortezomib in inhibiting the proteasome activity.

However, the Bortezomib inhibitory effect on overall proteolytic activity was several-fold greater when the proteasome assemblies were harvested and analyzed at earlier time-points. Without ruling out the possibility that FaDu cells may have evolved canonical mechanisms of drug resistance (*e.g.*, drug secretion and/or detoxification, or selective downregulation of 19S subunits^81^), the resistance of these cells to Bortezomib-induced apoptosis, which is in sharp contrast with the complete early proteasome inhibition after 2 h, can be likely explained through two different and not mutually exclusive hypotheses, namely: *i)* Bortezomib, being a reversible inhibitor, is displaced from the β5 catalytic site at a higher rate than in other cells; *ii)* among all cells employed in this study, FaDu are those which more readily synthesize de novo proteasome assemblies. Hypothesis *i)* indeed reflects a chemical property of Bortezomib which contributes to the resistance through which the cells can bypass the drug-induced death. However, although speculative at this stage, we envisage that hypothesis *ii)* may be of greater relevance to explain the observed behaviour for two main reasons:

- FaDu cells were the only cells clearly inducing the formation of alternative proteasome assemblies at 12 h and 24 h (but not at 20 min or 2 h), which clearly resembles non-canonical complexes, such as PA28-20S, as previously proposed to occur as an adaptative response to proteasome inhibition in experimental models other than those herein discussed^41^.
- FaDu cells showed the greatest extent of proteasome loss during treatment, a phenomenon which, to the best of our knowledge, has never been reported in the presence of a proteasome inhibitor. Remarkably, this Bortezomib-induced effect was observed, though to a variable extent, in all human cell lines tested so far, underscoring that it may be a general issue of pharmacological relevance, if confirmed in vivo.

At this stage, we cannot rule out a priori that it is a technical artifact, such as, for instance, epitope masking in the presence of undigested poly-ubiquitinylated substrates and further studies are demanded to clarify the biological relevance of this point.

Overall, our in vitro results indicated that the anti-cancer activities of Bortezomib was dependent on the type of HNC cell line, being positively related to the extent of proteasome inhibition exerted by Bortezomib in the different HNC cell lines. In fact, we have shown that the functional impairment of proteasome modulated in turn the signaling pathways involved in HNC progression. For example, Bortezomib had no effect on EGFR, ErbB2, JNK, p38 or AKT in FaDu cells. These effects paralleled the low efficacy of Bortezomib in inhibiting the proteasome activity in those cancer cells. Thus, our in vitro findings suggest that in HNC, showing limited proteasome resistance to Bortezomib and simultaneous upregulation of ErbB receptors-mediated signaling, anti-ErbB receptors antibodies or inhibitors of the ErbB receptors intrinsic tyrosine kinase activity should be used in combination with proteasome inhibitors. Furthermore, since other studies highlighted the development of different mechanisms of Bortezomib resistance in HNC, the investigation of the efficacy of combined treatment with Bortezomib and other inhibitors of different signaling pathways should be envisaged^13,27,55,56,59,82^.

In addition, regarding the analysis of the effects of Bortezomib on experimental models in HNC, there are several studies which have shown that Bortezomib has promising anticancer activities in mouse tumor models^45,46,56,83–85^. However, only two studies analyzed the ability of Bortezomib to counteract the in vivo HNC tumor growth, but they were restricted to xenografts implanted human larynx or tongue squamous cell carcinoma cell lines^46,56^. Accordingly, none of them have investigated the in vivo effect of Bortezomib in salivary gland carcinoma cell line. Thus, this is the first study showing the in vitro and in vivo growth inhibitory properties of Bortezomib in a salivary gland carcinoma cell line (SALTO-5) and the in vivo effects of Bortezomib on tumor-infiltrating immune cells, tumor vessels density, expression of ErbB2, AKT and cleaved caspase 3 and molecules involved in proteasome structural composition in SALTO-5 cell line transplanted in BALB-*neu*T mice. The Bortezomib effects on SALTO-5 cells were first analyzed in vitro, and we observed apoptosis and inhibition of ErbB2, p38 and AKT, and activation of JNK in Bortezomib-treated SALTO-5 cell line. In contrast to the human cells analyzed, Bortezomib induced an increase of ERK2 phosphorylation in SALTO-5 cells, which was still associated with the activation of the apoptotic process. On the other hand, regarding the effect of Bortezomib on proteasome, SALTO-5 cells displayed a significant recovery of proteasome particles after 24 h and of proteasome subunits content associated to a decrease of poly-ubiquitinated proteins. Although the study of apoptosis pathway did not put in evidence any resistance of these cells greater than that of SCC-15, A-253 or CAL-27 cells, it is likely that this mechanisms of resistance to the drug may emerge over a prolonged time of treatment. Cytotoxic and apoptotic effects of Bortezomib in SALTO-5 cell line were also observed by ultrastructural analysis.

In light of the in vitro results, we also evaluated the in vivo anti-tumor effects of i.p. administration of Bortezomib (0.5 mg/kg, twice a week) on tumor growth in BALB-*neu*T mice subcutaneously inoculated with syngeneic murine SALTO-5 cells. It has been reported that i.p. administration, at least twice a week, resulted in greater Bortezomib activity with less toxicity^86^. We confirmed the absence of Bortezomib toxicity in organs collected from Bortezomib-treated mice. The preclinical investigations have collectively demonstrated the anticancer activity of Bortezomib when used as monotherapy or in combination with chemotherapy, radiotherapy, or other anti-neoplastic agents^86–91^. Our results showed for the first time that Bortezomib reduced the growth of SALTO-5 murine cells in mice and increased the survival of the mice. In addition, the histological examination of tumors from Bortezomib-treated mice showed extensive necrosis and presence of apoptotic cells, as compared to the control mice. One previous study showed that tumor specimens, from mice transplanted with a human larynx cell line and treated with Bortezomib, displayed cell nuclear condensation and tissue degradation, as well as apoptotic areas^56^. According to the in vitro results, we provided evidence that Bortezomib inhibited in vivo the expression of ErbB2 simultaneously to that of AKT in tumors. It has to be highlighted that the effect on ErbB2 expression is very likely to have a strong impact on cancer cell growth. In fact, ErbB2 can dimerize with other ErbB receptors expressed by tumor cells, thus conferring a proliferative advantage of cells over homodimerization-induced growth. AKT inhibition by Bortezomib in vivo was previously observed in homogenates from tumors of a tongue squamous cell carcinoma cell line transplanted in mice^46^. Furthermore, we showed that Bortezomib induced an increased number of tumor-infiltrating immune cells, as indicated by the increase in both T and B lymphocytes, macrophages, and NK cells in transplanted (SALTO-5) cells from Bortezomib-treated mice. On the other hand, the increase of tumor-infiltrating immune cells in mice could be triggered by the large areas of cells necrosis induced by Bortezomib and therefore could represent an indirect effect of the drug. Nevertheless, the immune response elicited in the Bortezomib-treated mice could thus cooperate with the drug to inhibit tumor growth in vivo. In addition, we also investigated for the first time T cells activation and IFN-γ production of intratumoral and spleen lymphocytes from Bortezomib- or PBS + DMSO-treated mice transplanted with SALTO-5 cells. Our results showed that there was not a significant difference in the percentage of activated infiltrating lymphocytes and their IFN-γ production between Bortezomib- and DMSO-treated mice. This finding indicates that Bortezomib induces an increased number of tumor infiltrating T cells but not an increase of their functional activity.

We also reported, in agreement with a previous study^45^, that the inhibition of the tumor growth by Bortezomib was associated with a decrease in vessel density. For what concerns the in vivo tumor expression of proteasome subunits and ubiquitin, it is first worth underscoring that the immunostaining of two constitutive structural subunits, such as PSMA4 and PSMD4, was very faint in cancer cells of DMSO-treated animals. Interestingly, Bortezomib treatment induced a robust increase of immunostaining strengthening the hypothesis that the apparently low basal content of constitutive proteasome is critical for cancer cells. Thus, it could be due to a compensatory mechanism that the cells may have adopted to survive. Nevertheless, ubiquitin immunostaining was similar between the two groups of mice. Although ubiquitin staining often is a read-out of proteasome bulk proteolytic activity, it is worth underscoring that it is hard to discriminate between free ubiquitin (present at µmol/L concentration inside the cell) and ubiquitin conjugated to substrates. However, it can be likely stated that no accumulation of ubiquitin occurs in the presence of Bortezomib suggesting that compensatory mechanisms for the turnover of the natural substrates processed through the UPS take place. Unexpectedly, PSME1 expression, which encodes for the α subunit of PA28, was high in DMSO-treated cancer cells and very low in Bortezomib-treated cells^15^. The expression of PA28, an alternative regulatory particle which triggers 20S gate opening but has not ATP-dependent activity, is often induced in the presence of metabolic and pharmacological insults and it has been further reported as an adaptative mechanism of cancer cells treated with proteasome inhibitors *in vitro*^41^. Hence, this evidence raises some questions, that cannot be addressed at this stage, about the basal expression of proteasome sub-populations in this cancer and the metabolic and pharmacological implications this feature entails.

Overall, our results showed that anti-cancer activities of Bortezomib in tongue, pharynx and salivary gland cancer cells were dependent on cell line histotype and associated with the different extent of proteasome inhibition. The inhibition of proteasome was in turn associated with the modulation of the main signaling transduction pathways involved in HNC cellular transformation. Furthermore, for the first time we showed that Bortezomib displayed in vitro and in vivo antitumor activities in an adenocarcinoma of the salivary gland. The inhibition of tumor growth by Bortezomib was associated with tumor necrosis and apoptosis, with the simultaneous inhibition of ErbB2, AKT, and with the induction of a strong intratumoral immune response.

Our in vitro and in vivo findings further support the use of the proteasome inhibitor Bortezomib for the treatment of HNSCC and adenocarcinomas of the salivary gland and reveal its ineffectiveness in counteracting the activation of deregulated specific signaling pathways in HNC cell lines when resistance to proteasome inhibition is developed, thus suggesting the combined use of Bortezomib and specific drugs targeting signaling transduction pathways unaffected by Bortezomib treatment.

## Materials and methods

### Reagents

DMSO and Sulforhodamine B (SRB) were purchased from Sigma-Aldrich (Milan, Italy). Bortezomib was purchased from Selleck Chemical (Munich, Germany). Z-VAD-FMK was obtained from Calbiochem (San Diego, CA, USA). Antibodies against Bax, Bcl-2, AKT, phospho-AKT, JNK/SAPK1, JNK/SAPK (pT183/pY185), p38a/SAPK2a, and p38 MAPK (pT180/pY182) were obtained from BD Pharmingen (BD Biosciences, San Jose, CA, USA). Antibodies against caspase 9, caspase 8, activated caspase 3 were obtained from Cell Signaling Technology (MA, United States). Antibodies against PARP-1 (F-2), ERK1/2 (C-14) and phospho-ERK (E-4) were obtained from Santa Cruz Biotechnology (CA, USA). Anti-ErbB2 and anti-EGFR antisera were provided by Dr. M. H. Kraus (University of Alabama, Birmingham, AL, USA). Antibodies against Beclin-1 and p62/SQSTM1 were obtained from Abcam (Cambridge, United Kingdom) and the anti-LC3 antibody was purchased from Novus Biologicals (Littleton, CO, USA). Rabbit polyclonal anti-actin, anti-tubulin, and the goat anti-mouse or -rabbit IgG peroxidase conjugated secondary antibodies were obtained from Sigma-Aldrich (Milan, Italy).

### Cell lines and treatments

Cell lines derived from HNCs of the tongue (SCC-15, CAL-27), pharynx (FaDu) or salivary gland (A-253) were from ATCC (Manassas, VA, USA) and maintained in RPMI containing 10% fetal bovine serum, 100 U/ml penicillin, and 100 μg/ml streptomycin. Neu-overexpressing salivary gland cancer cells (H-2^d^) (SALTO-5) were kindly provided by Prof. F. Cavallo (University of Torino) and Prof. P. L. Lollini (University of Bologna) and kept in DMEM containing 20% fetal bovine serum (FBS). SALTO-5 cells were established from salivary carcinoma arising in BALB-*neu*T transgenic male mice hemizygous for the p53^172R-H^ transgene driven by the whey acidic protein promoter^92^. Bortezomib was dissolved in DMSO. For treatments, cells were incubated for the indicated times in the presence of Bortezomib (dose range 6.25–100 nM) or vehicle control (DMSO ≤ 0.1).

### Sulforhodamine B (SRB) assay

SRB assay was used to investigate cell proliferation, by measuring the cellular protein content of adherent cultures. SRB is a dye which stoichiometric binds to basic aminoacids under mild acidic conditions and dissociates using basic conditions^93^. Cells were seeded at 5 × 10^3^ cells/well in 96-well plates and incubated at 37° C to allow cell attachment. After 24 h, the medium was changed and cells were incubated for 24, 48 and 72 h with Bortezomib (6.25–100 nM) or with DMSO (amount equivalent to that administered at the highest concentration of Bortezomib). Cells were then fixed with cold trichloroacetic acid (final concentration 10%) for 1 h at 4° C. The assay was then performed as previously described^94^. Briefly, after four washes with distilled water, the plates were air-dried and stained for 30 min with 0.4% (wt/vol) SRB in 1% acetic acid. After four washes with 1% acetic acid to remove the unbound dye, the plates were air-dried and cell-bound SRB was dissolved with 200 µl/well of 10 mM unbuffered Tris Base solution. The optical density (O.D.) of the samples was determined at 540 nm using a spectrophotometric plate reader. The percentage survival of the cultures treated with Bortezomib was calculated by normalization of their O.D. values to those of the control cultures treated with DMSO^95^. The experiments were performed in triplicate and repeated three times.

### Trypan blue exclusion assay

For trypan blue exclusion assay, cells were seeded at 5 × 10^4^/well in 24-well plates and incubated at 37 °C to allow cells attachment. After 24 h, the medium was changed and cells were incubated for 24, 48, and 72 h with Bortezomib (6.25–100 nM) or DMSO. Thereafter, adherent as well as suspended cells of each well were harvested and stained with trypan blue (Sigma-Aldrich, Milan, Italy) and counted under a light optical microscope^96^. The experiments were repeated three times, and the percentage of dead cells was compared with the total number of cells^97^.

### FACS analysis

Asynchronized log-phase growing cells (60% confluent, approximately 2.5 × 10^5^ cells/well in 6-well plates) were treated with Bortezomib or with DMSO in a complete culture medium. Z-VAD-FMK was used at a final concentration of 40 µM for 2 h before addition of Bortezomib. After 48 h, adherent and suspended cells were harvested, centrifuged at 1500 rpm for 10 min and washed twice with cold phosphate buffered saline (PBS). The assay was then performed as previously described^98^. Cells were analyzed by flow cytometry using a FACS-Calibur cytometer running CellQuest Pro 5.2 software (BD Biosciences, San Jose, CA, USA).

### Western blotting

About 1 × 10^6^ cells were seeded in 100 mm tissue culture dishes 24 h prior to the addition of 12.5 (SCC-15 and CAL-27) or 25 nM (FaDu, A-253, SALTO-5) of Bortezomib or the vehicle control. After 24 h (for SCC-15) and 48 h of treatment, cells were harvested, washed twice with cold PBS, and lysed in RIPA buffer as previously described^99^. For immunoblotting analysis, 15–80 μg of cell lysates (depending on the experimental setting) were resolved in 10% SDS-PAGE and then transferred to nitrocellulose membranes^100^. Equal loading and transfer of proteins was verified by Ponceau red staining of the membranes and by analyzing actin expression. The assay was then performed as previously described^101^. A densitometric analysis of autoradiographic bands was performed with the ImageJ 1.53e software (NIH, MD, USA) after blot scanning and expressed as bar graphs in the figures.

### Native gel electrophoresis

Crude cell extracts (e.g., soluble fraction of the cell cytosol) were extracted under non-denaturing conditions through freeze-thawing cycles in 250 mM sucrose, 20% glycerol, 25 mM Tris–HCl, 5 mM MgCl_2_, 1 mM EDTA, 1 mM DTT, 2 mM ATP, pH 7.4. Thereafter, lysates were cleared by centrifugation at 13,000 rpm, 20 min, 4 °C and the protein concentration was determined by Bradford assay. For each experimental condition, 75 µg of proteins were separated under native conditions employing 3.5% acrylamide gel. Gels were then harvested in a clean dish and soaked in the reaction buffer (50 mM Tris, 5 mM MgCl_2_, 1 mM ATP, pH 7.5), which had been supplemented with 75 µM 7-amino-4-methylcoumarin (AMC) labeled Suc—Leu—Leu—Val—Tyr -AMC peptide (referred to as LLVY-AMC) (Boston Biochem, Boston, USA), a highly specific fluorogenic substrate of the proteasome chymotrypsin-like activity. This enzymatic proteolytic activity, which has been proven to be linearly correlated with the light intensity, was then recorded through a gel-documentation system (excitation λ = 365 nm; emission λ = visible)^102^. Proteins were then transferred to a HyBond-ECL nitrocellulose filters and probed with an antibody which recognizes an epitope shared by α1-7 subunits, but not by α4 (hereafter referred to as pan-α-subunits) (Protein-tech Group, Manchester, UK), diluted 1:3000 in 0.02% Tween-PBS fat-free milk, and then incubated with a Horseradish Peroxidase-conjugated anti-rabbit or anti-mouse IgG antibody (Biorad, Hercules, CA, USA), diluted 1:50,000 in 0.2% Tween-PBS fat-free milk.

### Treatment of BALB-*neu*T mice with Bortezomib

Transgenic BALB-*neu*T male mice were mated with BALB/c females (H-2^d^; Charles River, Calco, Italy) in the animal facilities of Tor Vergata University. Founder male BALB-*neu*T mice were kindly provided by Prof. G. Forni and Prof. F. Cavallo (University of Torino, Italy)^103^. Progenies were confirmed for the presence of the transgene by PCR^104^. BALB-*neu*T mice were subcutaneously injected in the right flank with a 0.2 ml suspension containing 1 × 10^6^ SALTO-5 cells in phosphate-buffered saline (PBS). Groups of BALB-*neu*T mice (8 mice per group) were treated i.p. with Bortezomib (0.5 mg/kg in 400 µl PBS + DMSO, twice a week) or with vehicle only (400 µl PBS + DMSO, twice a week) one week after the SALTO-5 tumor challenge. Mice were sacrificed at the first signs of distress^105^. In addition, another group of mice (n = 3) were treated i.p. with Bortezomib (0.5 mg/kg in 400 µl PBS + DMSO, twice a week) for 4 weeks to assess the toxicity of the drug. After 4 weeks, liver, lung, kidney, heart, and spleen were collected from these mice for histological examination after hematoxylin/eosin staining using 3 μm thick paraffin sections. The investigation has been conducted in accordance with the ethical standards and according to the Declaration of Helsinki and the ARRIVE guidelines. The work was conducted with the formal approval of the local [“Organismo Preposto al Benessere degli animali” (O.P.B.A.), University of Rome Tor Vergata] and national (Ministry of Health) animal care committees and animal experiments have been registered as legislation requires (Authorization from the Ministry of Health no. 844_2018-PR). A veterinary surgeon was present during the experiments. Animal care, before and after the experiments, was carried out only by trained personnel.

### Analysis of antitumor activity in vivo

Tumor growth was monitored weekly until tumor-bearing mice were sacrificed when the tumor exceeded a 20 mm width by cervical dislocation. Tumors were measured by a caliper in two perpendicular dimensions, and the volumes were calculated using the formula: width^2^ x length/2^97,106^.

### Histological analysis of tumors from mice treated with Bortezomib by optical microscopy

At sacrifice, tumors from three animals from each group of mice were used for histological examination after hematoxylin/eosin staining using 3 μm thick paraffin sections. Necrotic areas were measured using ImageJ 1.53e software on 10 microscopic fields. IHC was used to analyze the presence of caspase 3-positive cells (apoptotic cells) and the expression of ErbB2, AKT and phospho-AKT in tumors from PBS + DMSO- and Bortezomib-treated mice^107^. For IHC, antigen retrieval was performed on 3 μm thick paraffin sections using EDTA citrate, pH 7.8, or citrate pH 6.0 buffers for 30 min at 98 °C. Sections were then incubated for 1 h at room temperature [polyclonal anti-cleaved caspase3 and anti-ErbB2 antibodies; anti-CD8: clone YTS169.4 (Thermo Fisher Scientific, Waltham, MA, USA); anti-CD4: clone GK1.5 (Thermo Fisher Scientific, Waltham, MA, USA); anti-CD20: clone OTI4B4 (Novus Biologicals, Centennial, CO, USA); anti-F480: clone CI:A3-1 (BioXcell, Lebanon, NH, USA); anti-CD25: clone IL2R.1 (Thermo Fisher Scientific, Waltham, MA, USA); polyclonal anti-CD57 (Thermo Fisher Scientific, Waltham, MA, USA); anti-CD34: clone EP373Y (Abcam, Cambridge, UK); polyclonal anti-PSMA4 (ProteinTech Group, Chicago, IL); polyclonal anti-PSMD4 (ProteinTech Group, Chicago, IL); polyclonal anti-PSME1 (Abcam, Cambridge, UK); polyclonal anti-ubiquitin (ProteinTech Group, Chicago, IL); polyclonal] or overnight at 4 °C (anti-AKT and anti-phospho-AKT) with primary antibodies. To remove non-specific binding, slides were washed using PBS/Tween 20, pH 7.6. Antibody-antigen binding was revealed by the Horseradish Peroxidase-3,3-diaminobenzidine (HRP-DAB) Detection Kit (UCS Diagnostic, Rome, Italy). The count of cleaved caspase 3-positive cells (apoptotic cells) was performed on 10 microscopic fields at 200 × magnification. Semiquantitative ErbB2, AKT and phospho-AKT expression in tumors derived from PBS + DMSO- and Bortezomib-treated mice was estimated at × 200 magnification in at least 10 fields by two investigators in a blind fashion. ErbB2, AKT and phospho-AKT expression levels (negative, 0; weakly positive, 1; moderately positive, 2; strongly positive, 3) were scored. IHC evaluation of CD8, CD4, CD20, F480, CD25, CD57 was evaluated by counting the number of positive cells on 5 high power fields (HPF) (20x) by two investigators in a blind fashion. IHC evaluation of CD34 was evaluated by counting the number of positive vessels on 5 high power fields (HPF) (20x) by two investigators in a blind fashion. A combined scoring system was used for the IHC evaluation of PSMA4, PSMD4, PSME1 and ubiquitin. Specifically, the total score (0–6) was obtained by adding the score associated to the number of positive cells and the score related to the signal intensity. The score associated to the number of positive cells was defined as follow: 0 (1 ≤ Positive cells/HPF), 1 (2 ≤ x ≤ 10 Positive cells/HPF), 2 (11 ≤ x ≤ 20 Positive cells/HPF), 3 (≥ 21 Positive cells/HPF). The score related to the signal intensity was defined as follow: 0 (absent/very low Intensity/HPF), 1 (low Intensity/HPF), 2 (moderate Intensity/HPF), 3 (high Intensity/HPF). The interobserver reproducibility was > 95%. Sections were observed and photographed by Olympus BX53 light microscope or Zeiss Axioscope 5^98,106,108^.

### Cell extraction from mice tumor and spleen tissues and flow cytometry assay

At 5 weeks after SALTO transplantation, four hours after the last Bortezomib treatment, tumors from three animals from each group of mice were collected. To extract tumor-infiltrating lymphocytes (TIL), mouse tumors were mechanically dissociated in PBS 2% FBS onto a MACS SmartStrainers (70 µm) in a Petri dish, and a single cell suspension was obtained. Then leukocytes were enriched through 40/80 Percoll (GE Healthcare) density gradient, collecting cells at the interface between 40 and 80% Percoll solutions. Splenocytes were obtained by mechanical dissociation, followed by incubation with Red Blood Cell Lysis Buffer (Roche) for 10 min at room temperature for erythrocyte lysis^109^. Cells (10^6^) were stained with Fixable Viability Dye eFluor780 (eBioscience), and the following antibodies were used: CD4 FITC (clone GK1.5, eBioscience), CD49b PE (clone DX5, BD Biosciences), CD69 PECy7 (clone H1.2F3, BD Biosciences), CD3 AF647 (clone 17A2, BD Biosciences), CD8a BrilliantViolet785 (clone 53–6.7, BioLegend). Cells were fixed/permeabilized with Cytofix/Cytoperm solution according to the manufacturer’s instructions (BD Biosciences), and finally stained with IFN-γ BrilliantViolet421 (XMG1.2, BioLegend). Acquisition of 50,000 cells/sample in the lymphocytes’ gate for TIL and spleens were performed on a CytoFLEX flow cytometer (Beckman Coulter). Samples were analyzed by FlowJo software (version 10.8; Tree Star Inc.).

### Transmission electron microscopy

Ultrastructural analysis was performed on SALTO-5 cells treated with Bortezomib (12.5 nM for 24 h) or with DMSO. After treatment, cells were fixed in 2.5% glutaraldehyde in PBS pH 7.4, and the samples were processed for ultrastructural analysis following routine procedures and observed by a Morgagni 268D transmission electron microscopy^110^.

### Statistical analysis

The percentage of cell survival, different phases of the cell cycle and of cell death were preliminarily verified using the Kolmogorov–Smirnov test, and the data sets were analyzed by one-way analysis of variance (ANOVA) followed by the Newman-Keuls test. Differences in the intensity of immunoreactive bands were evaluated by a two-tailed Student’s t-test or one-way ANOVA followed by Tukey’s post-hoc significance test. Values with *p* ≤ 0.05 were considered significant. Survival curves and tumor volumes were analyzed using the Kaplan–Meier method and compared with a log-rank test (Mantel-Cox). Differences in tumor volumes were regarded as significant when the *p-value* was ≤ 0.05. Differences in the IHC score were evaluated by a two-tailed Student’s t test. Values with *p* ≤ 0.05 were considered significant.

## Supplementary Information

Supplementary Information.

## References

[1] Chow LQM (2020). Head and neck cancer. N. Engl. J. Med..

[2] Bray F (2018). Global cancer statistics 2018: GLOBOCAN estimates of incidence and mortality worldwide for 36 cancers in 185 countries. CA Cancer J. Clin..

[3] Joshi NP, Broughman JR (2021). Postoperative management of salivary gland tumors. Curr. Treat. Options Oncol..

[4] Bashraheel SS, Domling A, Goda SK (2020). Update on targeted cancer therapies, single or in combination, and their fine tuning for precision medicine. Biomed. Pharmacother..

[5] Lee YT, Tan YJ, Oon CE (2018). Molecular targeted therapy: treating cancer with specificity. Eur. J. Pharmacol..

[6] Kitamura N (2021). Current trends and future prospects of molecular targeted therapy in head and neck squamous cell carcinoma. Int. J. Mol. Sci..

[7] Nadhan R, Srinivas P, Pillai MR (2020). RTKs in pathobiology of head and neck cancers. Adv. Cancer Res..

[8] Alsahafi E (2019). Clinical update on head and neck cancer: molecular biology and ongoing challenges. Cell Death Dis..

[9] Harsha C (2020). Targeting AKT/mTOR in oral cancer: mechanisms and advances in clinical trials. Int. J. Mol. Sci..

[10] Vermorken JB (2008). Platinum-based chemotherapy plus cetuximab in head and neck cancer. N. Engl. J. Med..

[11] Byeon HK, Ku M, Yang J (2019). Beyond EGFR inhibition: multilateral combat strategies to stop the progression of head and neck cancer. Exp. Mol. Med..

[12] Picon H, Guddati AK (2020). Mechanisms of resistance in head and neck cancer. Am. J. Cancer Res..

[13] Roeten MSF, Cloos J, Jansen G (2018). Positioning of proteasome inhibitors in therapy of solid malignancies. Cancer Chemother. Pharmacol..

[14] Kisselev AF, Van der Linden WA, Overkleeft HS (2012). Proteasome Inhibitors: an expanding army attacking a unique target. Chem. Biol..

[15] Tundo GR (2020). The proteasome as a druggable target with multiple therapeutic potentialities: cutting and non-cutting edges. Pharmacol. Ther..

[16] Hershko A, Ciechanover A, Varshavsky A (2000). Basic medical research award. The ubiquitin system. Nat. Med..

[17] Livneh I, Cohen-Kaplan V, Cohen-Rosenzweig C, Avni N, Ciechanover A (2016). The life cycle of the 26S proteasome: from birth, through regulation and function, and onto its death. Cell Res..

[18] Groll M (2000). A gated channel into the proteasome core particle. Nat. Struct. Biol..

[19] Raynes R, Pomatto LC, Davies KJ (2016). Degradation of oxidized proteins by the proteasome: distinguishing between the 20S, 26S, and immunoproteasome proteolytic pathways. Mol. Aspects Med..

[20] Jackson G, Einsele H, Moreau P, Miguel JS (2005). Bortezomib, a novel proteasome inhibitor, in the treatment of hematologic malignancies. Cancer Treat. Rev..

[21] Papandreou CN, Logothetis CJ (2004). Bortezomib as a potential treatment for prostate cancer. Cancer Res..

[22] Kane RC, Bross PF, Farrell AT, Pazdur R (2003). Velcade: U.S. FDA approval for the treatment of multiple myeloma progressing on prior therapy. Oncologist.

[23] Adams J (2002). Proteasome inhibitors as new anticancer drugs. Curr. Opin. Oncol..

[24] Jagannath S (2005). Bortezomib therapy alone and in combination with dexamethasone for previously untreated symptomatic multiple myeloma. Br. J. Haematol..

[25] Kondagunta GV (2004). Phase II trial of bortezomib for patients with advanced renal cell carcinoma. J. Clin. Oncol..

[26] Shah SA (2001). 26S proteasome inhibition induces apoptosis and limits growth of human pancreatic cancer. J. Cell. Biochem..

[27] Chung CH (2010). Nuclear factor-kappa B pathway and response in a phase II trial of bortezomib and docetaxel in patients with recurrent and/or metastatic head and neck squamous cell carcinoma. Ann. Oncol..

[28] Dudek AZ (2009). Phase I study of bortezomib and cetuximab in patients with solid tumours expressing epidermal growth factor receptor. Br. J. Cancer.

[29] Davies AM (2007). Phase I study of two different schedules of bortezomib and pemetrexed in advanced solid tumors with emphasis on non-small cell lung cancer. J. Thorac. Oncol..

[30] Gilbert J (2013). Phase II 2-arm trial of the proteasome inhibitor, PS-341 (bortezomib) in combination with irinotecan or PS-341 alone followed by the addition of irinotecan at time of progression in patients with locally recurrent or metastatic squamous cell carcinoma of the head and neck (E1304): a trial of the Eastern Cooperative Oncology Group. Head Neck.

[31] Falchook GS (2014). Targeting hypoxia-inducible factor-1α (HIF-1α) in combination with antiangiogenic therapy: a phase I trial of bortezomib plus bevacizumab. Oncotarget.

[32] Argiris A (2011). A phase 2 trial of bortezomib followed by the addition of doxorubicin at progression in patients with recurrent or metastatic adenoid cystic carcinoma of the head and neck: a trial of the eastern cooperative oncology group (E1303). Cancer.

[33] Argiris A (2011). Early tumor progression associated with enhanced EGFR signaling with bortezomib, cetuximab, and radiotherapy for head and neck cancer. Clin. Cancer Res..

[34] Kubicek GJ (2012). Phase I trial using the proteasome inhibitor bortezomib and concurrent chemoradiotherapy for head-and-neck malignancies. Int. J. Radiat. Oncol. Biol. Phys..

[35] Bei R (2001). Colocalization of multiple ErbB receptors in stratified epithelium of oral squamous cell carcinoma. J Pathol.

[36] Rivas S, Gómez-Oro C, Antón IM, Wandosell F (2018). Role of Akt isoforms controlling cancer stem cell survival, phenotype and self-renewal. Biomedicines.

[37] Mizushima N, Yoshimori T (2007). How to interpret LC3 immunoblotting. Autophagy.

[38] Klionski DJ (2021). Guidelines for the use and interpretation of assays for monitoring autophagy (4th edition). Autophagy.

[39] Bowler E (2020). Pharmacological inhibition of ATR can block autophagy through an ATR-independent mechanism. iScience.

[40] Elsasser S, Schmidt M, Finley D (2005). Characterization of the proteasome using native gel electrophoresis. Methods Enzymol..

[41] Welk V (2016). Inhibition of proteasome activity induces formation of alternative proteasome complexes. J. Biol. Chem..

[42] Adams J (2003). The proteasome: structure, function, and role in the cell. Cancer Treat. Rev..

[43] Adams J (1999). Proteasome inhibitors: a novel class of potent and effective antitumor agents. Cancer Res..

[44] Shanker A (2008). Treating metastatic solid tumors with bortezomib and a tumor necrosis factor-related apoptosis-inducing ligand receptor agonist antibody. J. Natl. Cancer Inst..

[45] Sunwoo JB (2001). Novel proteasome inhibitor PS-341 inhibits activation of nuclear factor-κB, cell survival, tumor growth and angiogenesis in squamous cell carcinoma. Clin. Cancer Res..

[46] Lin YC, Chen KC, Chen CC, Cheng AL, Chen KF (2012). CIP2A-mediated Akt activation plays a role in bortezomib-induced apoptosis in head and neck squamous cell carcinoma cells. Oral Oncol..

[47] Li C, Johnson DE (2012). Liberation of functional p53 by proteasome inhibition in human papilloma virus-positive head and neck squamous cell carcinoma cells promotes apoptosis and cell cycle arrest. Cell Cycle.

[48] Ow TJ (2020). Apoptosis signaling molecules as treatment targets in head and neck squamous cell carcinoma. Laryngoscope.

[49] Li C, Li R, Grandis JR, Johnson DE (2008). Bortezomib induces apoptosis via Bim and Bik up-regulation and synergizes with cisplatin in the killing of head and neck squamous cell carcinoma cells. Mol. Cancer Ther..

[50] Bullenkamp J (2014). Bortezomib sensitises TRAIL-resistant HPV-positive head and neck cancer cells to TRAIL through a caspase-dependent, E6-independent mechanism. Cell Death Dis..

[51] Fribley A, Zeng Q, Wang CY (2004). Proteasome inhibitor PS-341 induces apoptosis through induction of endoplasmic reticulum stress-reactive oxygen species in head and neck squamous cell carcinoma cells. Mol. Cell. Biol..

[52] Kim J (2010). PS-341 and histone deacetylase inhibitor synergistically induce apoptosis in head and neck squamous cell carcinoma cells. Mol. Cancer Ther..

[53] Wagenblast J (2008). Effects of combination treatment of bortezomib and dexamethasone in SCCHN cell lines depend on tumor cell specificity. Oncol. Rep..

[54] Leinung M (2012). Fighting cancer from different signaling pathways: Effects of the proteasome inhibitor Bortezomib in combination with the polo-like-kinase-1-inhibitor BI2536 in SCCHN. Oncol. Lett..

[55] Li CV (2009). Bortezomib up-regulates activated signal transducer and activator of transcription-3 and synergizes with inhibitors of signal transducer and activator of transcription-3 to promote head and neck squamous cell carcinoma cell death. Mol. Cancer Ther..

[56] Chen Z (2008). Differential bortezomib sensitivity in head and neck cancer lines corresponds to proteasome, nuclear factor-kappaB and activator protein-1 related mechanisms. Mol. Cancer Ther..

[57] Wagenblast J, Hambek M, Baghi M, Knecht R (2008). Effect of bortezomib on EGFR expression in head and neck squamous cell carcinoma cell lines. Anticancer Res..

[58] Li C, Johnson DE (2012). Bortezomib induces autophagy in head and neck squamous cell carcinoma cells via JNK activation. Cancer Lett..

[59] Chang I, Wang CY (2016). Inhibition of HDAC6 protein enhances bortezomib-induced apoptosis in head and neck squamous cell carcinoma (HNSCC) by reducing autophagy. J. Biol. Chem..

[60] Weber CN, Cerniglia GJ, Maity A, Gupta AK (2007). Bortezomib sensitizes human head and neck carcinoma cells SQ20B to radiation. Cancer Biol. Ther..

[61] Di Villeneuve L, Souza IL, Tolentino FDS, Ferrarotto R, Schvartsman G (2020). Salivary gland carcinoma: novel targets to overcome treatment resistance in advanced disease. Front. Oncol..

[62] Vattemi E (2008). Systemic therapies for recurrent and/or metastatic salivary gland cancers. Expert Rev. Anticancer Ther..

[63] Andry G, Hamoir M, Locati LD, Licitra L, Langendijk JA (2012). Management of salivary gland tumors. Expert Rev. Anticancer Ther..

[64] Hong YS (2012). Bortezomib induces G2-M arrest in human colon cancer cells through ROS-inducible phosphorylation of ATM-CHK1. Int. J. Oncol..

[65] Ling YH (2003). Mechanisms of proteasome inhibitor PS-341-induced G(2)-M-phase arrest and apoptosis in human non-small cell lung cancer cell lines. Clin. Cancer Res..

[66] Lu G, Punj V, Chaudhary PM (2008). Proteasome inhibitor Bortezomib induces cell-cycle arrest and apoptosis in cell lines derived from Ewing’s sarcoma family of tumors and synergizes with TRAIL. Cancer Biol. Ther..

[67] Wang Y (2010). Targeted proteasome inhibition by Velcade induces apoptosis in human mesothelioma and breast cancer cell lines. Cancer Chemother. Pharmacol..

[68] Bei R (2004). Frequent overexpression of multiple ErbB receptors by head and neck squamous cell carcinoma contrasts with rare antibody immunity in patients. J. Pathol..

[69] Xia W (1999). Combination of EGFR, HER-2/neu, and HER-3 is a stronger predictor for the outcome of oral squamous cell carcinoma than any individual family members. Clin. Cancer Res..

[70] Graus-Porta D, Beerli RR, Daly JM, Hynes NE (1997). ErbB-2, the preferred heterodimerization partner of all ErbB receptors, is a mediator of lateral signaling. EMBO J..

[71] Lioni M (2008). Bortezomib induces apoptosis in esophageal squamous cell carcinoma cells through activation of the p38 mitogen-activated protein kinase pathway. Mol. Cancer Ther..

[72] Yan H (2007). Arsenic trioxide and proteasome inhibitor bortezomib synergistically induce apoptosis in leukemic cells: the role of protein kinase C delta. Leukemia.

[73] Tournier C (2013). The 2 faces of JNK signaling in cancer. Genes Cancer.

[74] Hideshima T (2003). Molecular mechanisms mediating antimyeloma activity of proteasome inhibitor PS-341. Blood.

[75] Chauhan D (2004). Targeting mitochondria to overcome conventional and bortezomib/proteasome inhibitor PS-341 resistance in multiple myeloma (MM) cells. Blood.

[76] Yang Y (2004). Proteasome inhibitor PS-341 induces growth arrest and apoptosis of non-small cell lung cancer cells via the JNK/c-Jun/AP-1 signaling. Cancer Sci..

[77] Kao C (2014). Bortezomib enhances cancer cell death by blocking the autophagic flux through stimulating ERK phosphorylation. Cell Death Dis..

[78] Periyasamy-Thandavan S (2010). Bortezomib blocks the catabolic process of autophagy via a cathepsin-dependent mechanism, affects endoplasmic reticulum stress and induces caspase-dependent cell death in antiestrogen-sensitive and resistant ER+ breast cancer cells. Autophagy.

[79] Armstrong JL (2011). Oncogenic B-RAF signaling in melanoma impairs the therapeutic advantage of autophagy inhibition. Clin Cancer Res.

[80] Restuccia DF, Hemmings BA (2010). From man to mouse and back again: advances in defining tumor AKTivities in vivo. Dis. Model. Mech..

[81] Tsvetkov P (2015). Compromising the 19S proteasome complex protects cells from reduced flux through the proteasome. Elife.

[82] Ruschak AM, Slassi M, Kay LE, Schimmer AD (2011). Novel proteasome inhibitors to overcome Bortezomib resistance. J. Natl. Cancer Inst..

[83] LeBlanc R (2002). Proteasome inhibitor PS-341 inhibits human myeloma cell growth in vivo and prolongs survival in a murine model. Cancer Res..

[84] Tan C, Waldmann TA (2002). Proteasome inhibitor PS-341, a potential therapeutic agent for adult T-cell leukemia. Cancer Res..

[85] Amiri KI, Horton LW, LaFleur BJ, Sosman JA, Richmond A (2004). Augmenting chemosensitivity of malignant melanoma tumors via proteasome inhibition: implication for bortezomib (VELCADE, PS-341) as a therapeutic agent for malignant melanoma. Cancer Res..

[86] Boccadoro M, Morgan G, Cavenagh J (2005). Preclinical evaluation of the proteasome inhibitor bortezomib in cancer therapy. Cancer Cell Int..

[87] Wang S (2017). Leucovorin enhances the anti-cancer effect of bortezomib in colorectal cancer cells. Sci. Rep..

[88] Adams J (2004). The development of proteasome inhibitors as anticancer drugs. Cancer Cell.

[89] Mitsiades N (2003). The proteasome inhibitor PS-341 potentiates sensitivity of multiple myeloma cells to conventional chemotherapeutic agents: therapeutic applications. Blood.

[90] Hideshima T (2001). The proteasome inhibitor PS-341 inhibits growth, induces apoptosis, and overcomes drug resistance in human multiple myeloma cells. Cancer Res..

[91] Ma MH (2003). The proteasome inhibitor PS-341 markedly enhances sensitivity of multiple myeloma tumor cells to chemotherapeutic agents. Clin. Cancer Res..

[92] Pannellini T (2006). Timely DNA vaccine combined with systemic IL-12 prevents parotid carcinomas before a dominant-negative p53 makes their growth independent of HER-2/neu expression. J. Immunol..

[93] Vichai V, Kirtikara K (2016). Sulforhodamine B colorimetric assay for cytotoxicity screening. Nat. Protoc..

[94] Masuelli L (2017). In vitro and in vivo anti-tumoral effects of the flavonoid apigenin in malignant mesothelioma. Front. Pharmacol..

[95] Benvenuto M (2016). In vitro and in vivo inhibition of breast cancer cell growth by targeting the Hedgehog/GLI pathway with SMO (GDC-0449) or GLI (GANT-61) inhibitors. Oncotarget.

[96] Strober W (2015). Trypan blue exclusion test of cell viability. Curr. Protoc. Immunol..

[97] Benvenuto M (2018). Effect of the BH3 mimetic polyphenol (−)-gossypol (AT-101) on the in vitro and in vivo growth of malignant mesothelioma. Front. Pharmacol..

[98] Masuelli L (2020). In vivo and in vitro inhibition of osteosarcoma growth by the pan Bcl-2 inhibitor AT-101. Invest. New Drugs.

[99] Masuelli L (2014). Resveratrol potentiates the in vitro and in vivo anti-tumoral effects of curcumin in head and neck carcinomas. Oncotarget.

[100] Alesiani D, Cicconi R, Mattei M, Bei R, Canini A (2009). Inhibition of Mek 1/2 kinase activity and stimulation of melanogenesis by 5,7-dimethoxycoumarin treatment of melanoma cells. Int. J. Oncol..

[101] Masuelli L (2017). Curcumin blocks autophagy and activates apoptosis of malignant mesothelioma cell lines and increases the survival of mice intraperitoneally transplanted with a malignant mesothelioma cell line. Oncotarget.

[102] Sbardella D (2018). The insulin-degrading enzyme is an allosteric modulator of the 20S proteasome and a potential competitor of the 19S. Cell. Mol. Life Sci..

[103] Rovero S (2000). DNA vaccination against rat her-2/Neu p185 more effectively inhibits carcinogenesis than transplantable carcinomas in transgenic BALB/c mice. J. Immunol..

[104] Frajese GV (2018). Electrochemically reduced water delays mammary tumors growth in mice and inhibits breast cancer cells survival in vitro. Evid. Based Complement. Alternat. Med..

[105] Masuelli L (2007). Gene-specific inhibition of breast carcinoma in BALB-*neu*T mice by active immunization with rat Neu or human ErbB receptors. Int. J. Oncol..

[106] Focaccetti C (2020). Curcumin enhances the antitumoral effect induced by the recombinant vaccinia neu vaccine (rV-neuT) in mice with transplanted salivary gland carcinoma cells. Nutrients.

[107] Masuelli L (2010). Local delivery of recombinant vaccinia virus encoding for neu counteracts growth of mammary tumors more efficiently than systemic delivery in neu transgenic mice. Cancer Immunol. Immunother..

[108] Benvenuto M (2015). Natural humoral immune response to ribosomal P0 protein in colorectal cancer patients. J. Transl. Med..

[109] Pacella I (2018). Wnt3a Neutralization Enhances T-cell responses through indirect mechanisms and restrains tumor growth. Cancer Immunol. Res..

[110] Masuelli L (2017). Chloroquine supplementation increases the cytotoxic effect of curcumin against Her2/neu overexpressing breast cancer cells in vitro and in vivo in nude mice while counteracts it in immune competent mice. Oncoimmunology.

