# Supplementary material for: Proteasome inhibition by bortezomib parallels a reduction in head and neck cancer cells growth, and an increase in tumor-infiltrating immune cells
Source: Sci Rep. 2021 Sep 24;11:19051. doi: 10.1038/s41598-021-98450-6 (PMC8463577; doi:10.1038/s41598-021-98450-6)
Supplement: Supplementary file 1 — Supplementary Information. [file 41598_2021_98450_MOESM1_ESM.docx]

Proteasome inhibition by bortezomib parallels a reduction in head and neck cancer cells growth, and an increase in tumor-infiltrating immune cells

Monica Benvenuto ^1,2, †^, Sara Ciuffa ^2, †^, Chiara Focaccetti ^2,3, †^, Diego Sbardella ^4^, Sara Fazi ^5^, Manuel Scimeca ^1,3,6^, Grazia Raffaella Tundo ^4^, Giovanni Barillari ^2^, Maria Segni ^7,8^, Elena Bonanno ^1,6,9^, Vittorio Manzari ^2^, Andrea Modesti ^2^, Laura Masuelli ^5^, Massimo Coletta ^2,4, ‡^ and Roberto Bei ^2, ‡,^ *

^1^ Saint Camillus International University of Health and Medical Sciences, via di Sant’Alessandro 8, 00131, Rome, Italy; [monica.benvenuto@unicamillus.org](mailto:monica.benvenuto@unicamillus.org) (M.B.); [manuel.scimeca@uniroma2.it](mailto:manuel.scimeca@uniroma2.it) (M.S); [elena.bonanno@uniroma2.it](mailto:elena.bonanno@uniroma2.it) (E.B)

^2^ Department of Clinical Sciences and Translational Medicine, University of Rome “Tor Vergata”, via Montpellier 1, 00133, Rome, Italy; sara.ciuffa3@gmail.com (S.C.); chiara.focaccetti@uniroma5.it (C.F.); [barillar@uniroma2.it](mailto:barillar@uniroma2.it) (G.B.); manzari@med.uniroma2.it (V.M.); modesti@med.uniroma2.it (A.M.); coletta@med.uniroma2.it (M.C.); bei@med.uniroma2.it (R.B.)

^3^  Department of Human Science and Promotion of the Quality of Life, San Raffaele University Rome, via di Val Cannuta 247, 00166, Rome, Italy

^4^  IRCCS-Fondazione Bietti, Rome, Italy; [diego.sbardella@fondazionebietti.it](mailto:diego.sbardella@fondazionebietti.it) (D.S.); [grazia.tundo@libero.it](mailto:grazia.tundo@libero.it) (G.R.T.)

^5^  Department of Experimental Medicine, University of Rome “Sapienza”, viale Regina Elena 324, 00161, Rome, Italy; sara.fazi@uniroma1.it (S.F.); laura.masuelli@uniroma1.it (L.M.)

^6^ Department of Experimental Medicine, "Tor Vergata" University of Rome, via Montpellier 1, 00133, Rome, Italy

^7^ Department of Maternal Infantile and Urological Sciences, University of Rome “Sapienza”, viale Regina Elena 324, 00161, Rome, Italy; maria.segni@uniroma1.it

^8^ Pediatric Endocrinology Unit, Policlinico Umberto I, viale Regina Elena 364, 00161, Rome, Italy

^9^ "Diagnostica Medica" & "Villa dei Platani", Neuromed Group, 83100, Avellino, Italy

* Correspondence: bei@med.uniroma2.it; Tel.: +39-0672596522

† These authors contributed equally to this work

**‡** These last authors contributed equally to this work

**SUPPLEMETARY INFORMATIONS**

**
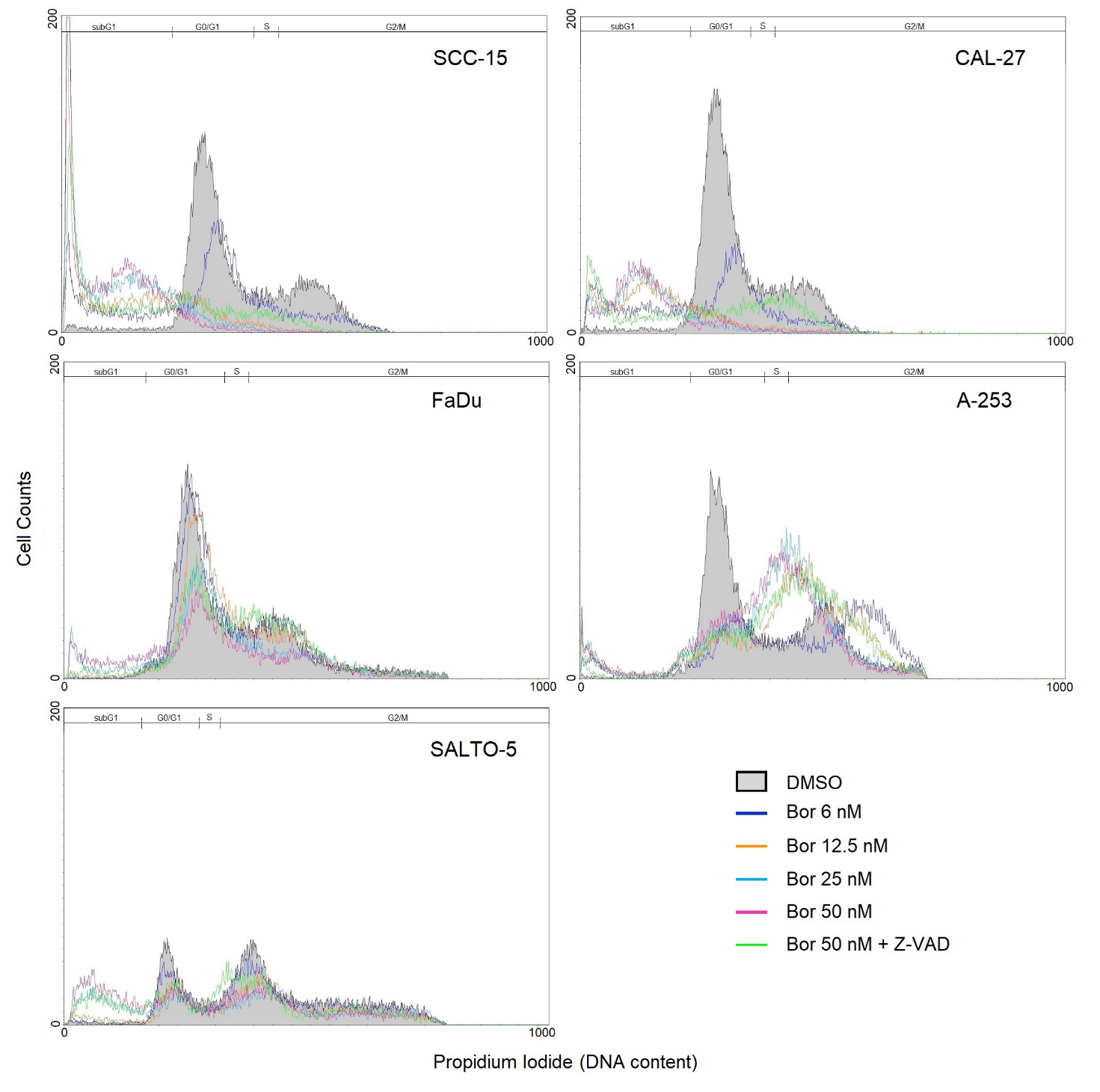
**

**Figure S1:** Effect of Bortezomib on cell cycle distribution.

FACS analysis of DNA content was performed on asynchronized log phase growing HNC cells treated for 48 hours with DMSO or Bortezomib (Bor) at 6.25-50 nM or Bortezomib at 50 nM + Z-VAD-FMK. A representative experiment is shown in the figure.


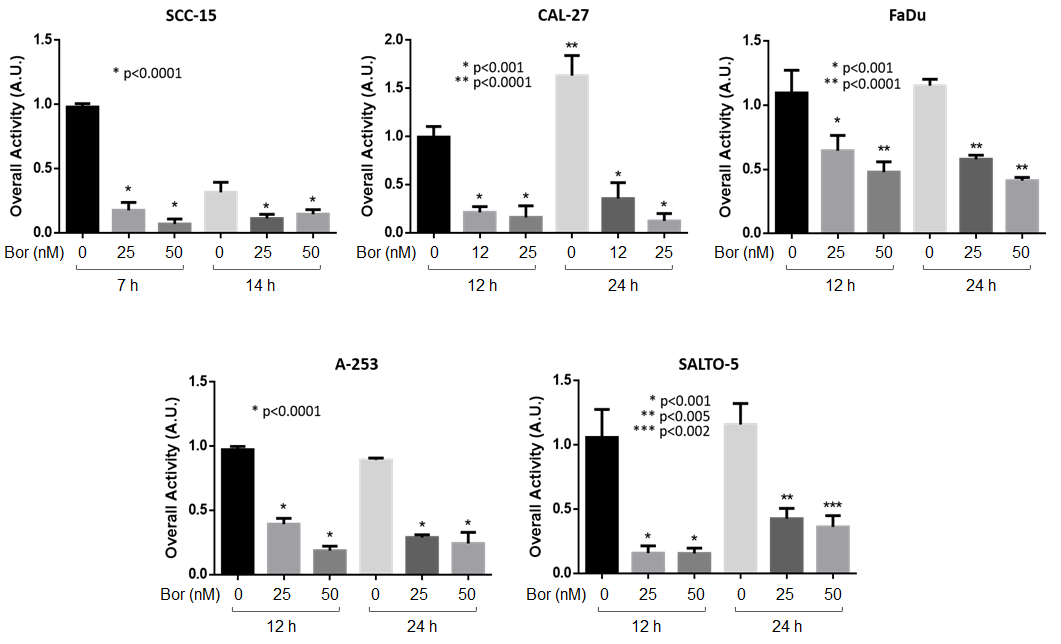


**Figure S2:** Semi-quantitative analysis of proteasome proteolytic activity.

Proteasome activity of particles shown in Figure 6a was determined by calculating the overall light intensity of all proteasome assemblies along the lane. A nominal value of 1 was assigned to the intensity of the untreated cell harvested at the earliest time-point; A representative panel of a single experiment is shown. Every assay has been carried out three independent times. Data are presented as mean±SD (n=3). One-way ANOVA followed by Tukey’s post-hoc significance test. The statistical analysis reported refers to the ratio between each Bortezomib-related experimental condition *vs* untreated cells at the same time-point.


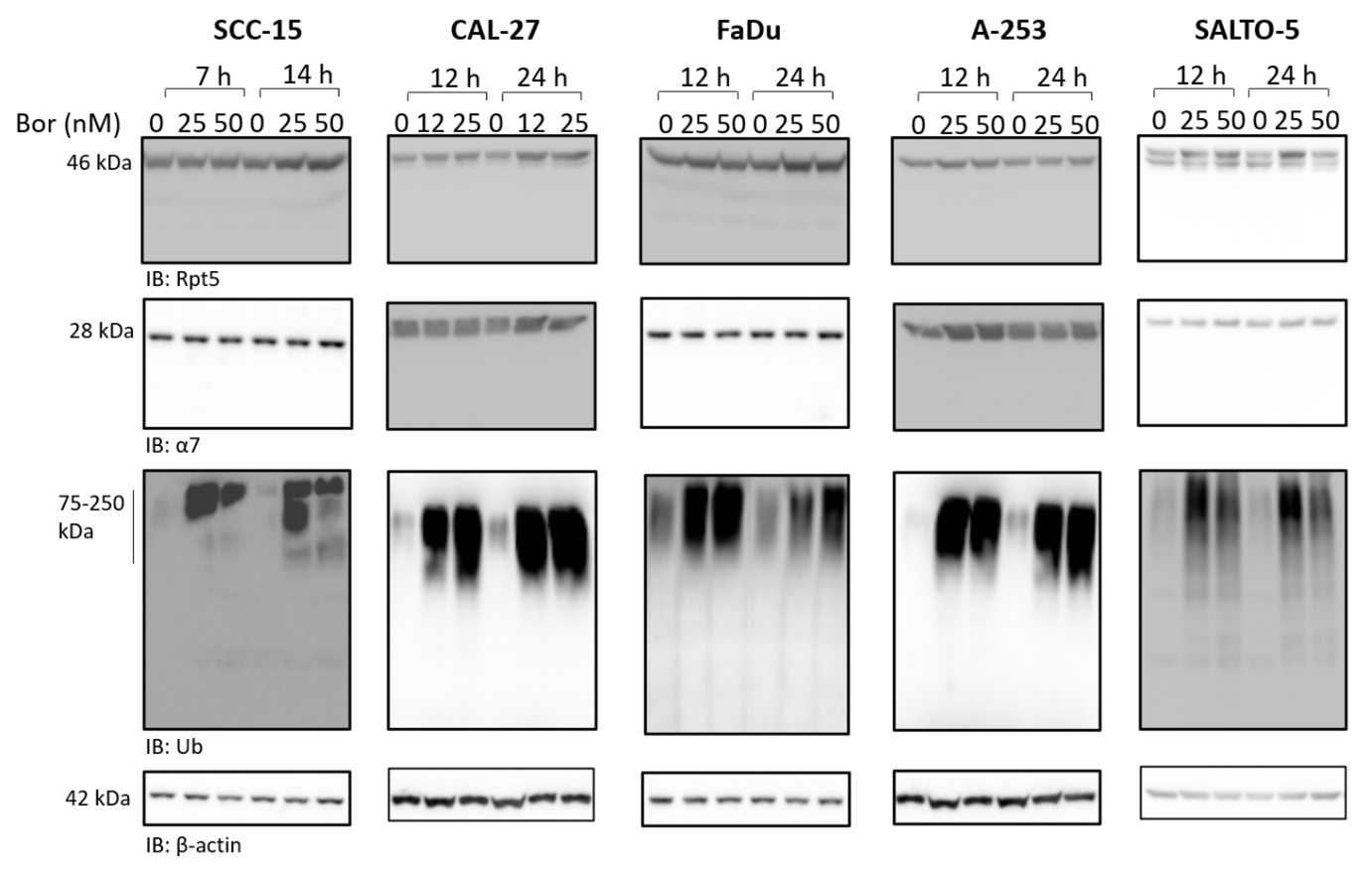


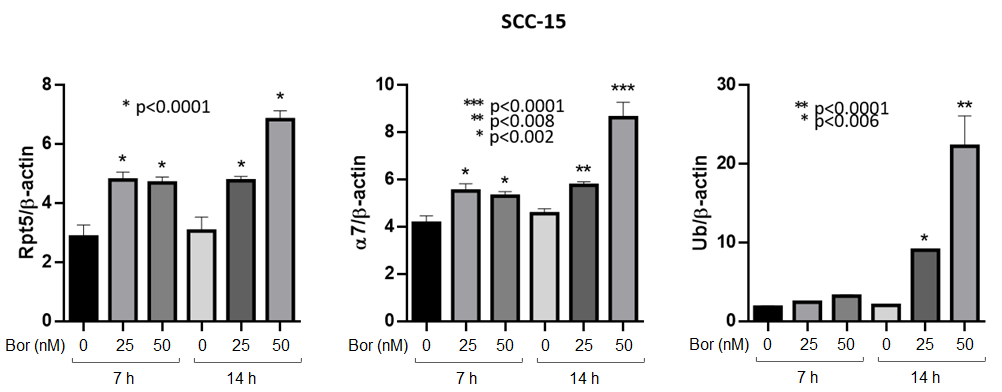


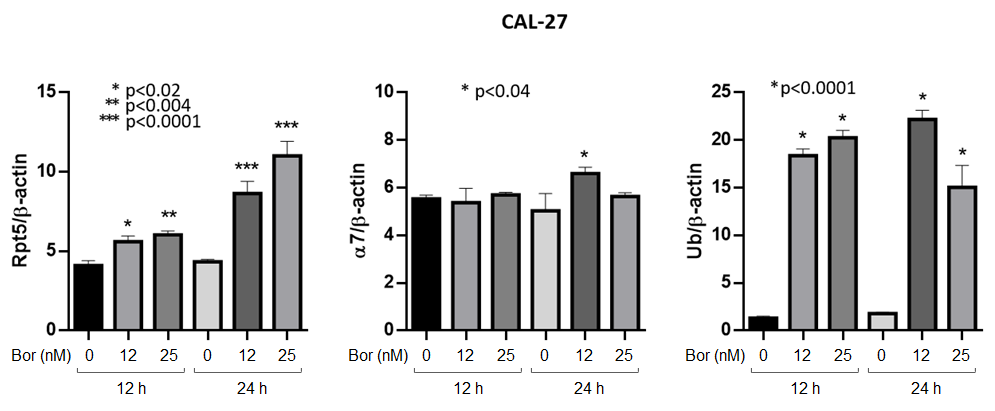


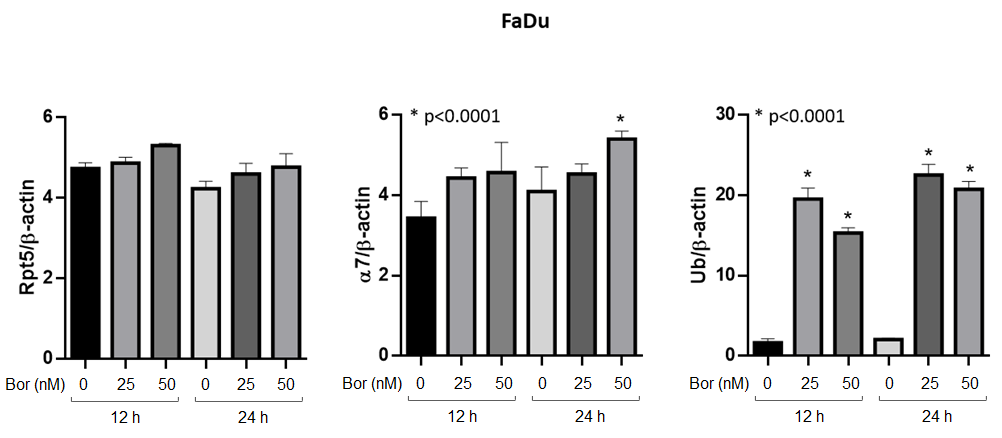


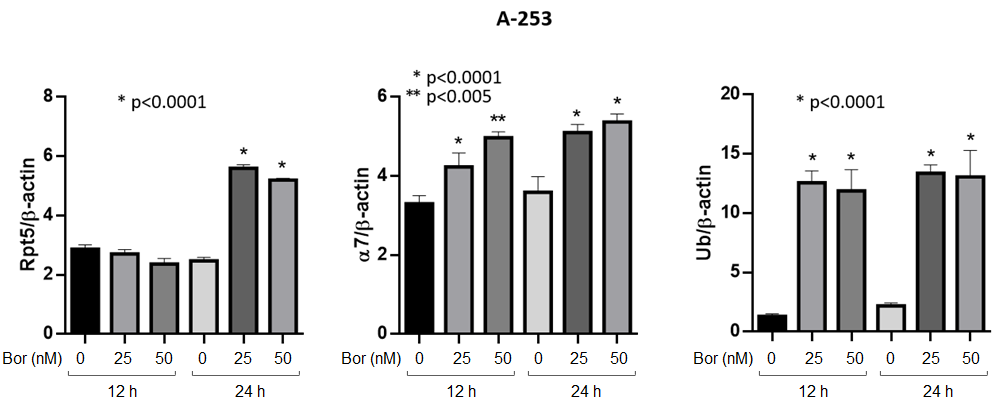


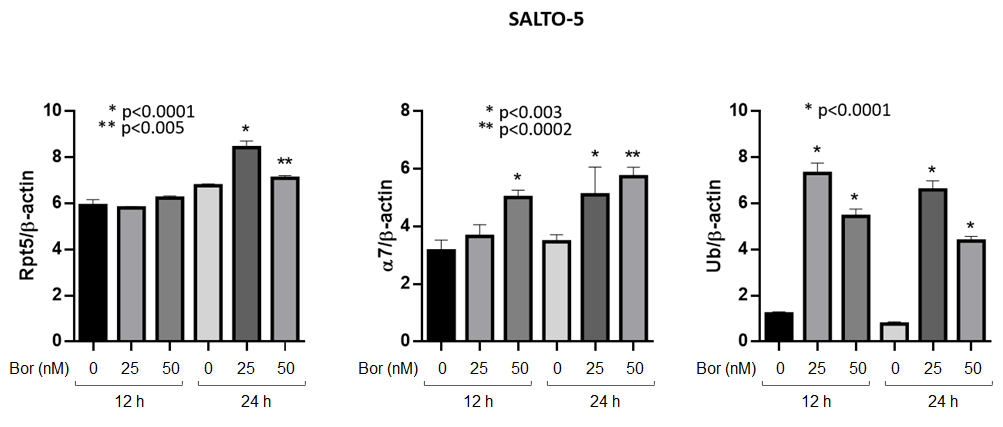


**Figure S3**: Semiquantitative analysis of selected proteasome subunits and of poly-ubiquitinated proteins.

Cytosolic extracts of cell lines were analysed for free α7, Rpt5 and poly-ubiquitinated proteins by denaturing and reducing Western Blotting. β-actin was used as internal control. A representative panel of a single experiment is shown. Every assay has been carried out three independent times. Densitometric analysis of immunoblotting data are presented as mean±SD (n=3). One-way ANOVA followed by Tukey’s post-hoc significance test. The statistical analysis reported refers to the ratio between each Bortezomib-related experimental condition *vs* untreated cells at the same time-point. Uncropped Western blots are reported in Supplementary Fig. S9.


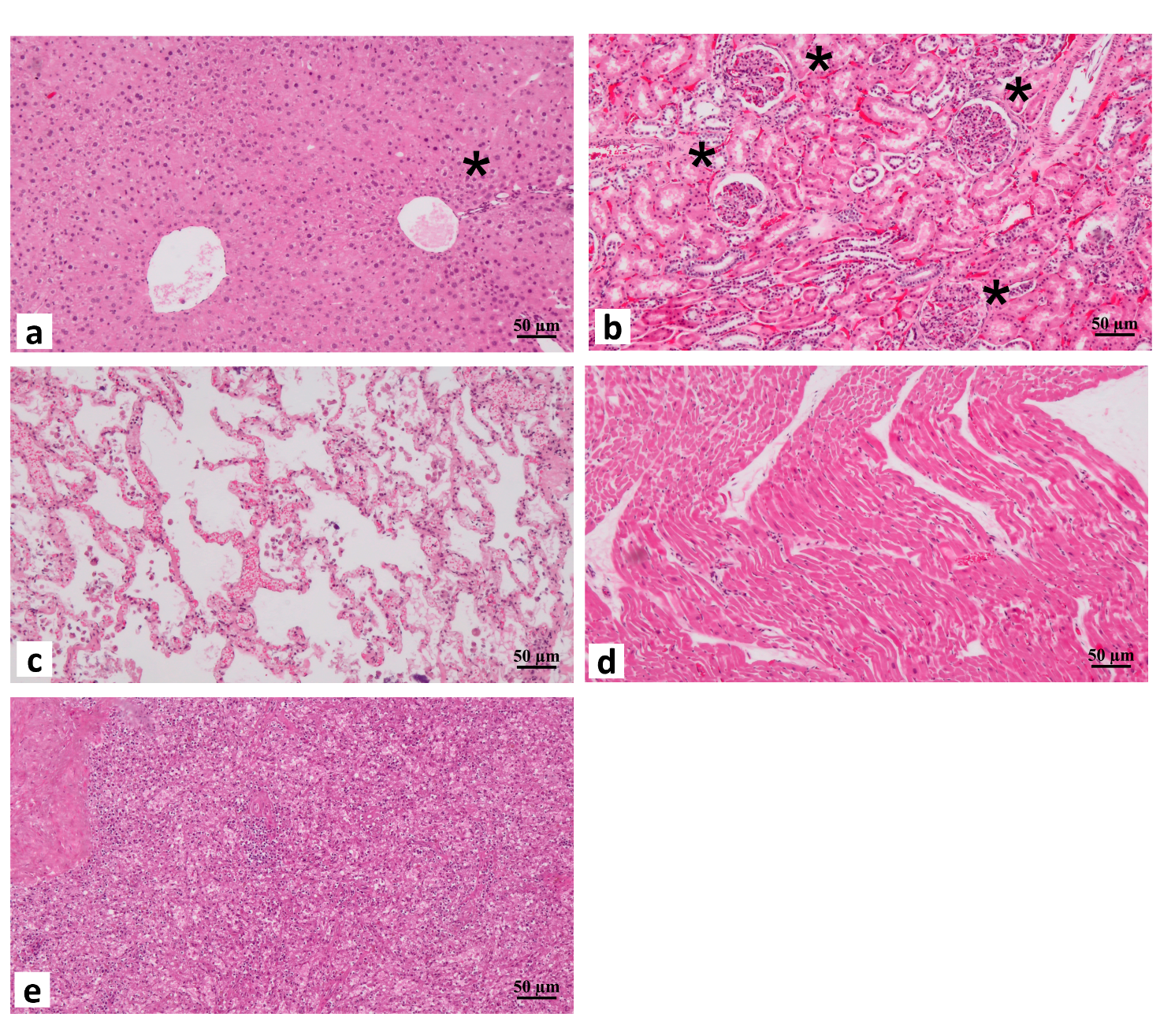


**Figure S4**: Toxicological evaluation of Bortezomib treatment by histological analysis.

**(a)** Hematoxylin and eosin stain shows a well-preserved liver parenchyma with a normal portal triad (asterisk). **(b)** Image displays no alterations in both renal glomerulus (asterisks) and tubules. **(c)** Well-preserved lung parenchyma in a mouse treated with Bortezomib. **(d)** No signal of hypertrophy and inflammation in the hearth of a mouse treated with Bortezomib. **(e)** Image shows normal architecture of a spleen of a mouse treated with Bortezomib. Scale bars correspond to 50 µm.


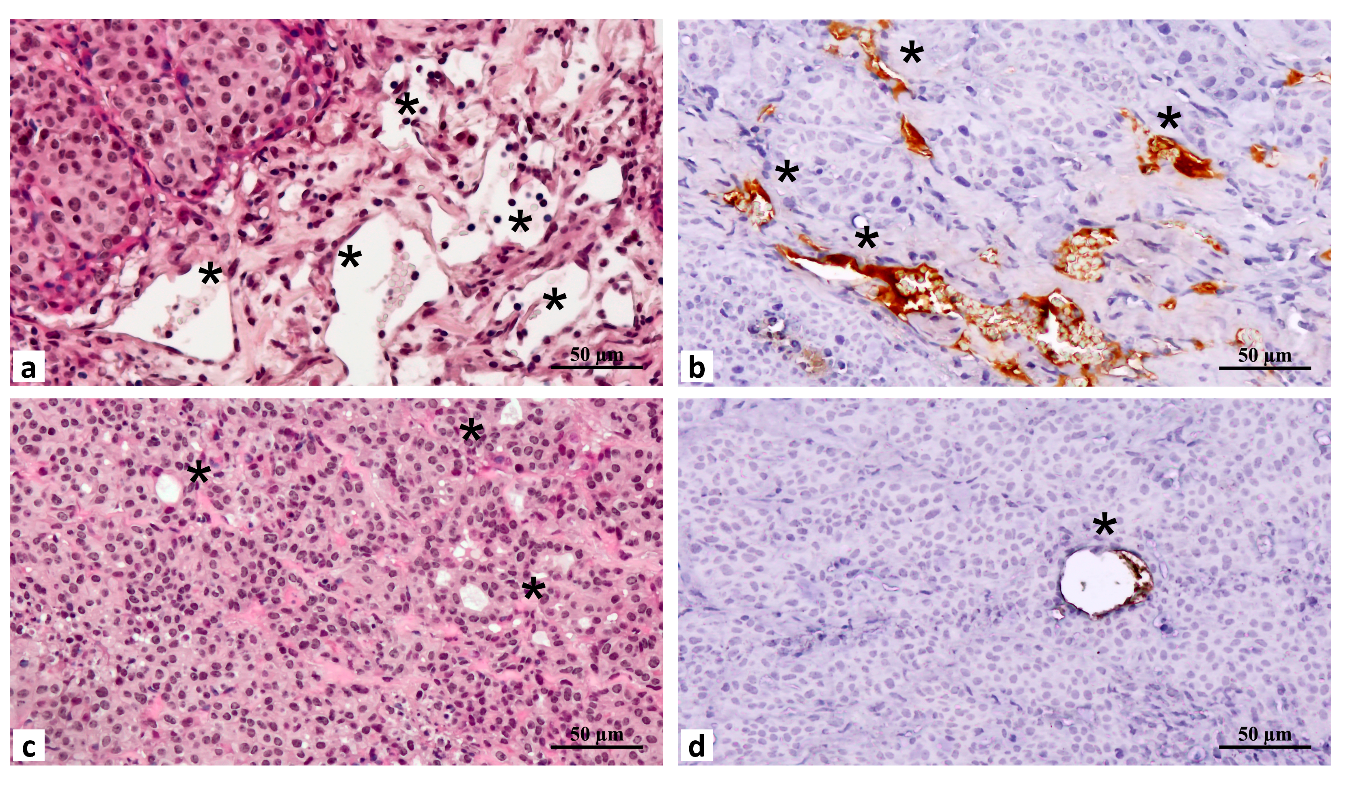


**Figure S5:** Characterization of tumor vessels. Histology and IHC analysis of tumors from Bortezomib-treated mice (Bor) or control-treated mice (PBS+DMSO). Tumor tissues from three mice in each group were analyzed and representative images were reported. **(a)** Hematoxylin and eosin stain shows numerous intra-tumoral large vessels in a control-treated sample (asterisks). **(b)** Several CD34^+^ large vessels in a control-treated tumor tissue (asterisks). **(c)** Bortezomib-treated tumor mass with small vessels (asterisks). **(d)** CD34^+^ staining reveals the presence of only one large vessels in a Bortezomib-treated tumor (asterisk). Scale bars correspond to 50 µm.





**Figure S6:** Analysis of tumor infiltrating lymphocytes in Bortezomib- and PBS+DMSO-treated mice.

**(a)** Representative dot plot of CD69-IFN-γ cell fractions in CD3^+^CD4^+^, CD3^+^CD8^+^ or CD3^-^CD49b^+^ lymphocytes collected from tumors (TUM) and spleens (SPL) of Bortezomib (Bor)- (n=3) or PBS+DMSO (DMSO)-treated (n=3) mice. **(b)** Cumulative stacked bar graph of CD69-IFN-γ cell fractions in CD3^+^CD4^+^, CD3^+^CD8^+^ or CD3^-^CD49b^+^ lymphocytes collected from tumors (TUM) and spleens (SPL) of Bortezomib (Bor)- or PBS+DMSO (DMSO)-treated mice.


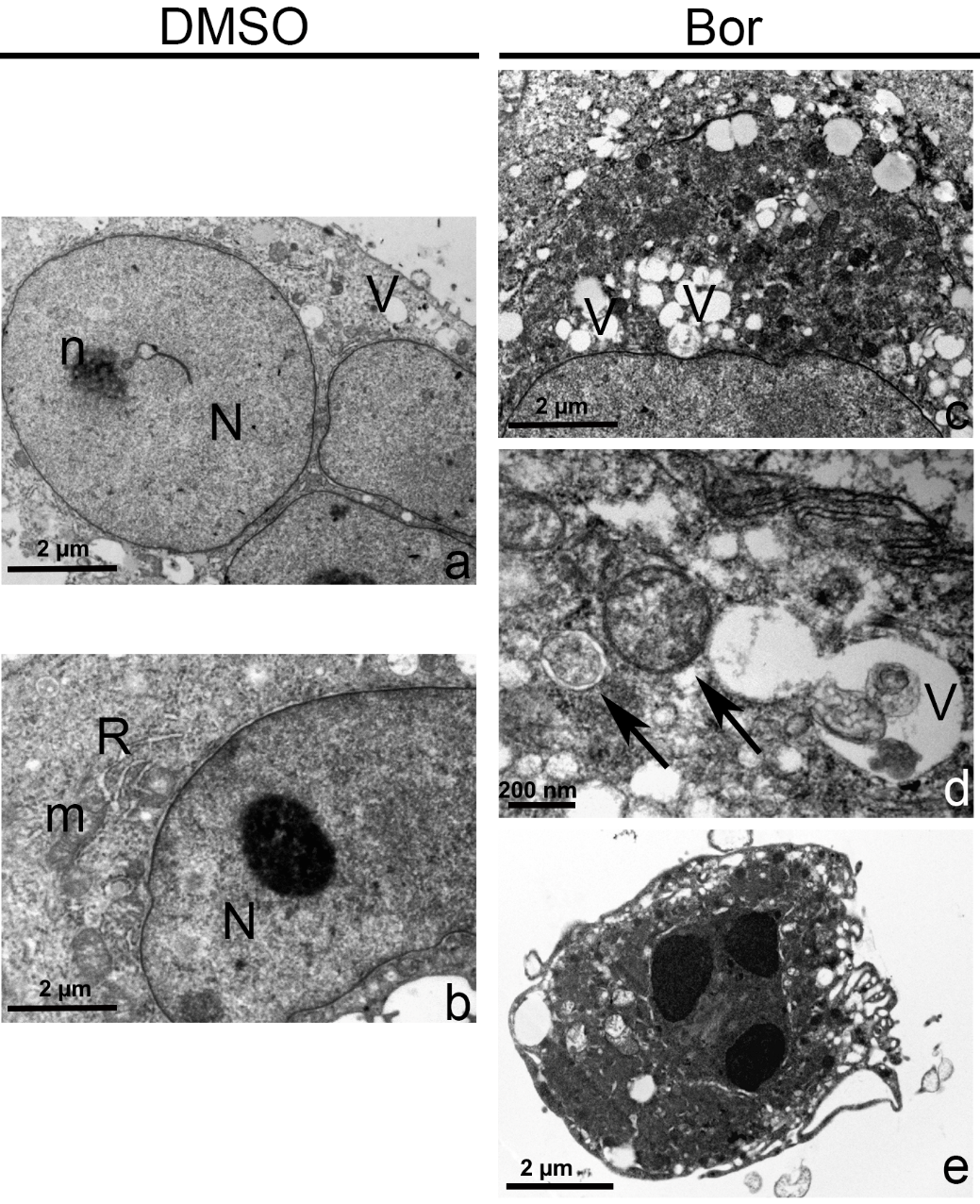


**Figure S7:** Ultrastructural analysis of Bortezomib- or DMSO-treated SALTO-5 cells.

Ultrastructural analysis of SALTO-5 cells treated with Bortezomib (Bor) at a concentration of 25 nM (c, d, e) or with DMSO (a, b), as a vehicle control, for 24 hours. The presence of numerous vacuoles surrounded by single membrane (V) or double membrane resembling autophagosomes (arrows) in Bor-treated cells was observed. Apoptotic cells were also visible (e). N: nucleus; n: nucleolus; m: mitochondria; R: endoplasmic reticulum; V: vacuoles. Scale bars correspond to 2 µm (a,b,c,e) and 200 nm (d).

**
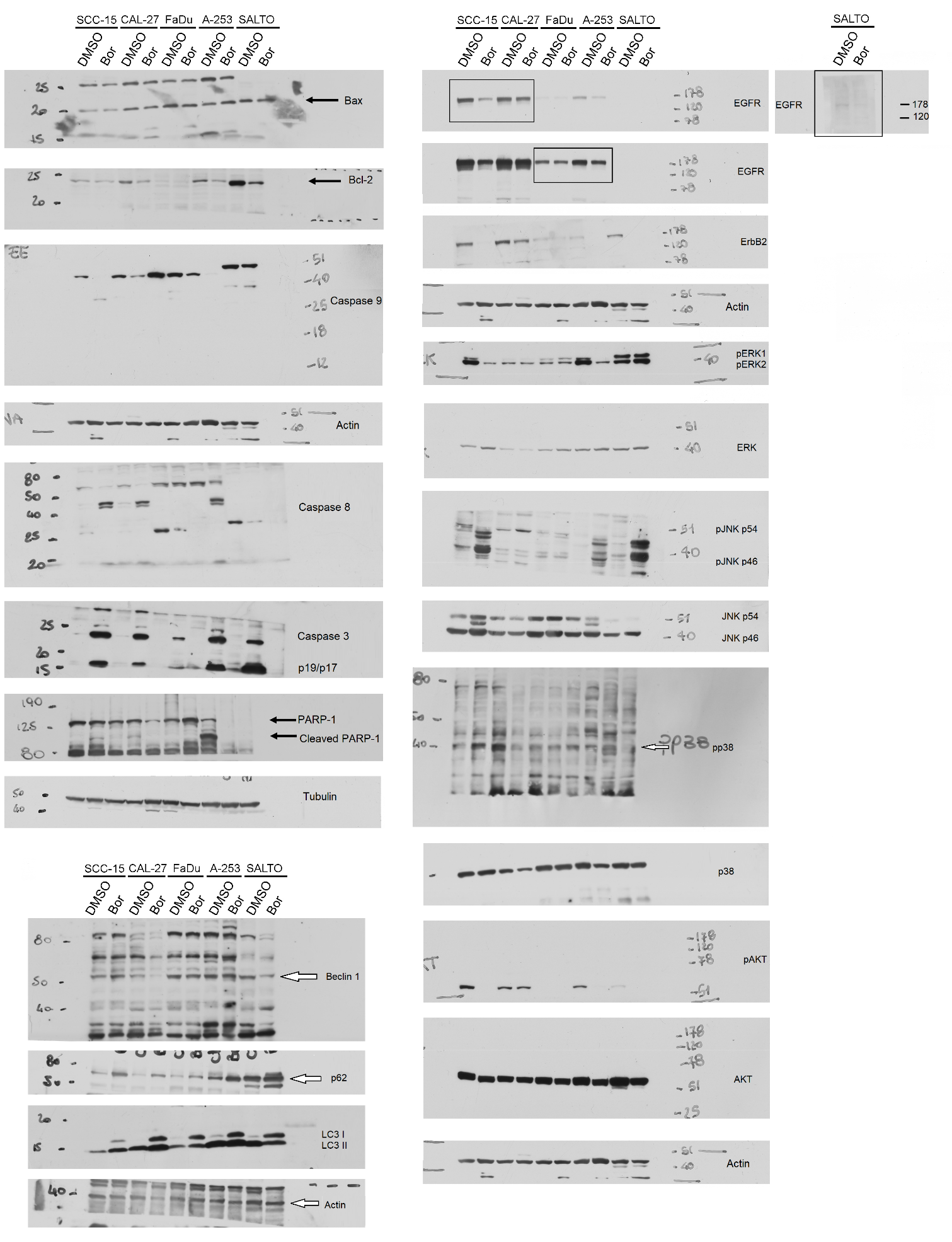
**

**Figure S8:** Uncropped Western blots corresponding to Figures 3, 4, 5. Note that the membranes were cut before probing.


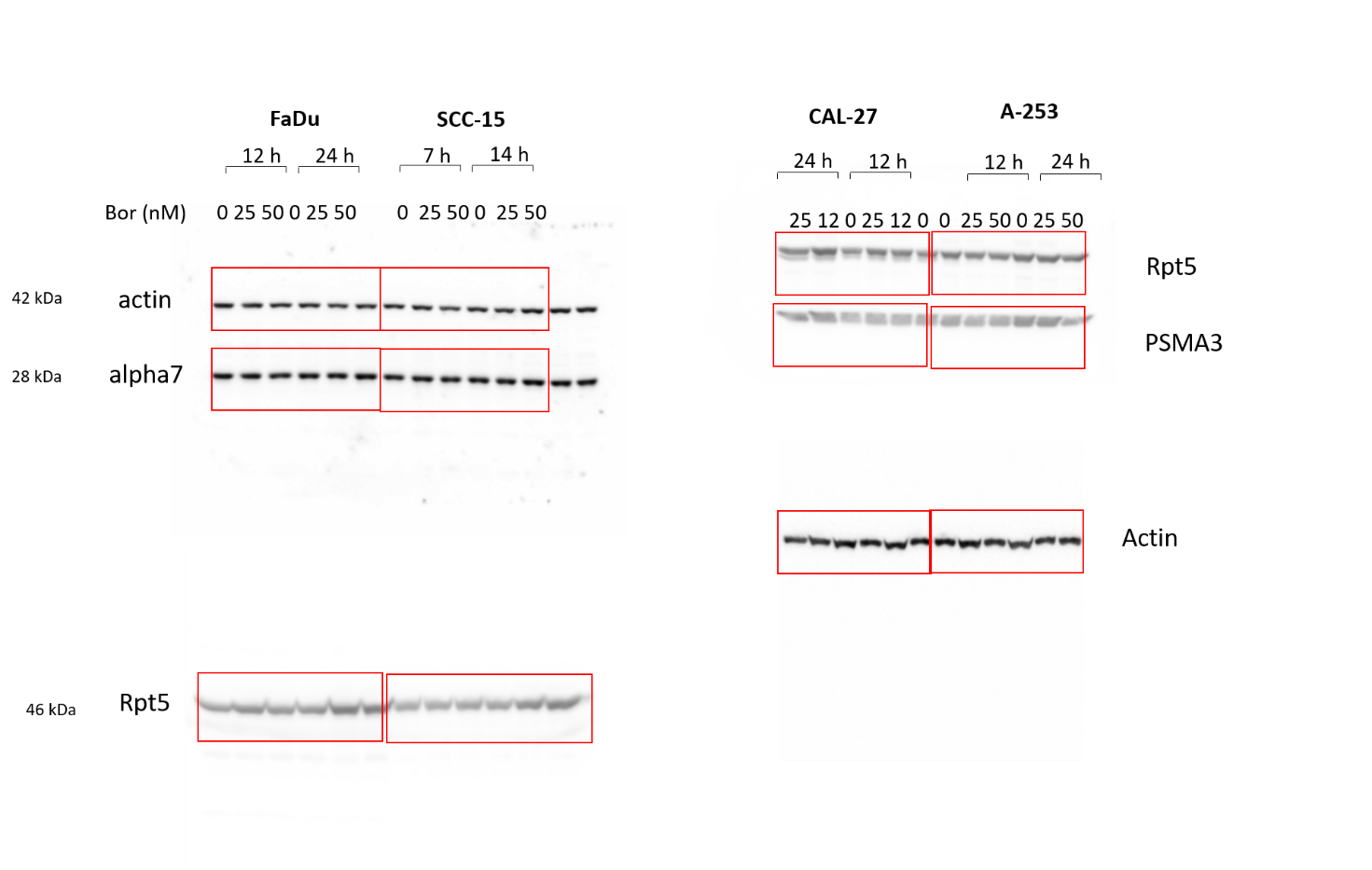


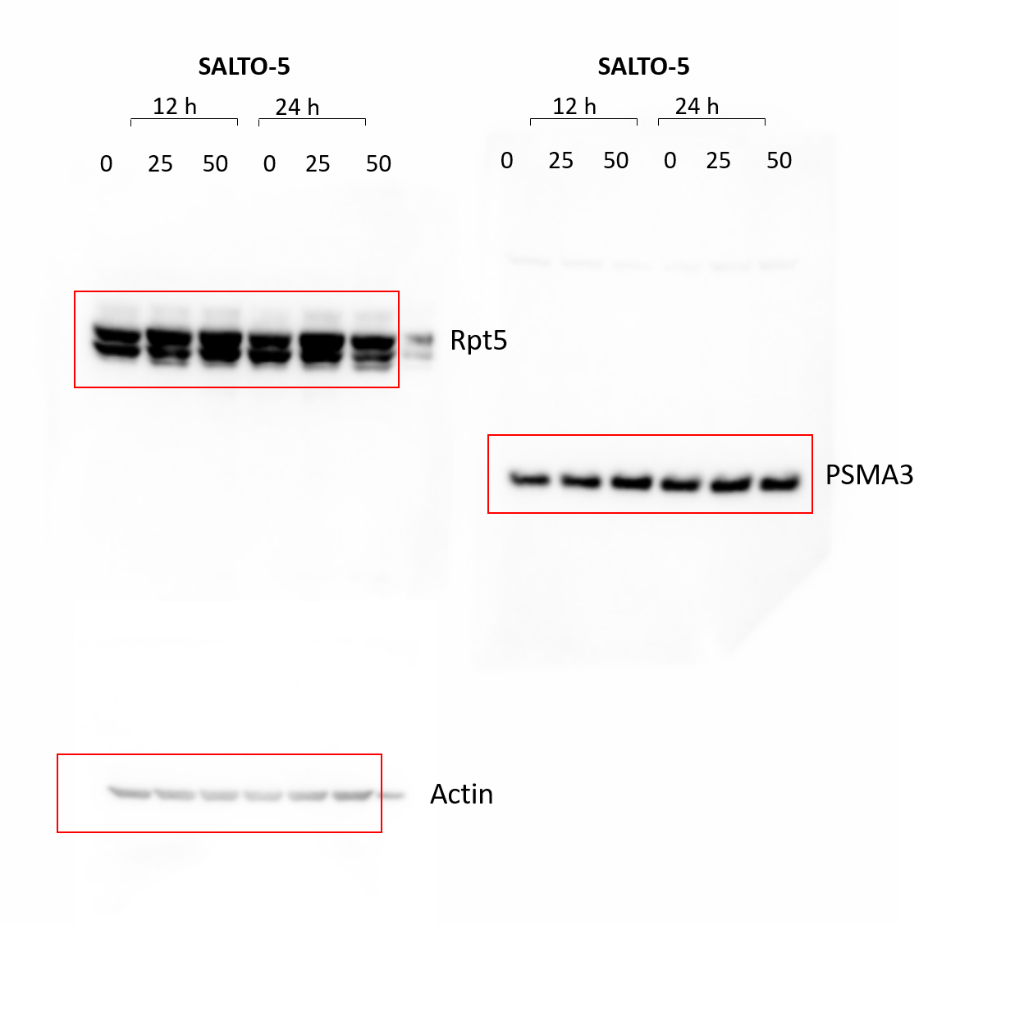


**Figure S9:** Uncropped Western blots corresponding to proteasome subunits expression in Supplementary Fig. S3.
